# Necropsy Technique and Histological Characterisation of Organs From Neonatal Puppies: What Do We Know?

**DOI:** 10.1002/vms3.70392

**Published:** 2025-05-13

**Authors:** Victória Ghedin, Jéssica Cardia de Melo, Fernanda Barthelson Carvalho de Moura, Keylla Helena Nobre Pacífico Pereira, Carlos Mario Gonzalez Zambrano, Juliana Jurado Jiménez, Gabriela Abreu Botelho, Luíz Guilherme Decore Benevenuto, Reiner Silveira de Moraes, Fernando Carmona Dinau, Pedro Pol Ximenes, Ana Júlia Motta da Costa, Natália Freitas de Souza, Maria Clara Boni Raffi, Tatiana Pessoa Onuma, Natália Camargo Faraldo, Maria Lucia Gomes Lourenço, Sérgio Luis Felisbino, Tatiane Terumi Negrão Watanabe, Monica Barthelson Carvalho de Moura, Luis Mauricio Montoya‐Flórez, Francisco Pedraza‐Ordoñez, Noeme Sousa Rocha

**Affiliations:** ^1^ São Paulo State University (UNESP) School of Veterinary Medicine and Animal Science Department of Veterinary Clinics Botucatu Brazil; ^2^ Federal University of Alagoas (UFAL) Department of Veterinary Medicine Viçosa – Alagoas Brazil; ^3^ São Paulo State University (UNESP) Faculty of Medicine, Department of Pathology Botucatu Brazil; ^4^ São Paulo State University (UNESP) School of Veterinary Medicine and Animal Science Department of Veterinary Surgery and Reproduction Botucatu Brazil; ^5^ São Paulo State University (UNESP) Institute of Biosciences, Morphology Department Botucatu Brazil; ^6^ Antech Diagnostics Mars Petcare Science & Diagnostics Fountain Valley California USA; ^7^ São Leopoldo Mandic Campinas Unit Campinas Brazil; ^8^ Universidad Nacional de Colombia (UNAL) Faculty of Veterinary Medicine Department of Animal Health Bogotá Colombia; ^9^ Laboratorio de Patología Veterinaria Universidad de Caldas Manizales Colombia

**Keywords:** canine neonatology, histology, histopathology, newborn dog, postmortem examination

## Abstract

Neonatal mortality in dogs is high, ranging from approximately 5% to 35%, yet investigations into its causes and risk factors remain scarce. Postmortem examination is a crucial tool for identifying the underlying causes of neonatal death and improving disease diagnosis. However, the anatomical, physiological and histological differences between neonates and adults present unique challenges for necropsy procedures. Establishing a standardised neonatal necropsy protocol is essential for accurately determining the causes of death and associated diseases. This pilot study aimed to standardise a necropsy technique for canine neonates. During the examination, the spleen, heart, lungs, tongue, stomach, intestines, liver, pancreas, kidneys, bladder, brain and bones were collected from nine neonatal canines. The method involved monoblock organ removal, followed by separation on the basis of the standard organisation used in adult necropsies. Histological staining and analysis revealed structural differences between neonatal and adult tissues. Neonatal hepatocytes presented a more basophilic cytoplasm, suggesting high metabolic activity, whereas gastric glands and parietal cells were less developed, indicating ongoing maturation. Additionally, the pancreas, lungs, kidneys and spleen displayed morphological characteristics consistent with those of the early development stages. Understanding these particularities may enhance veterinary diagnostic and clinical approaches, contribute to the development of preventive measures and ultimately reduce neonatal mortality in dogs. The standardisation of necropsy protocols facilitates the recognition of disease patterns, improves pathological documentation and supports further research on neonatal canine mortality.

## Graphical abstract

The mortality rate of dogs during pregnancy is high.

A postmortem examination allows the identification of possible causes of puppy mortality.

Newborns present anatomical, physiological and histological characteristics.

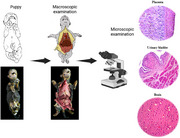

## 1 Introduction

Neonatal mortality represents a challenge for veterinarians and kennel breeders, with global rates ranging from 5.7% to 35% (Mila et al. 2017). Primary causes of mortality include maternal diseases, complications during delivery, infectious diseases and congenital malformations. However, the specific causes of death and associated risk factors are often not thoroughly investigated (Lamm and Njaa 2012, Pereira et al. 2022).

Necropsy is a valuable diagnostic tool for identifying the underlying causes of puppy mortality (Coelho 2002, Löhr 2011, Munnich 2008). Through a detailed examination of the cadaver and its organs, conventional necropsy aids in disease diagnosis by detecting pathological alterations. This technique, which has been practised since ancient times with refinements over the years, allows the assessment of animal management practices, the implementation of preventive measures and the control of zoonotic diseases and contributes to both clinical research and legal enforcement (De Souza et al. 2018, Estevam et al. 2022, King et al. 2013, Mila et al. 2021, Nobre Pacifico Pereira et al. 2019).

Despite its importance, few studies have histologically characterised neonatal canine organs, which is considered crucial for accurate histopathological interpretation. (Mila et al. 2021, Sorribas 2013, Sorribas 2013) Therefore, this study aimed to outline the key steps involved in neonatal postmortem examination and to provide anatomical and histological characterisation of neonatal organs.

## 2 Materials and Methods

### 2.1 Location and Animals

The animals used in this study were obtained from the Service of Small Animal Reproduction and sent to the Veterinary Pathology Laboratory. The evaluation was performed on nine neonates, with an average of 30 days of age, from different breeds. Each animal died during the neonatal period and was subjected to postmortem examination between April and December 2021. Patients who were aborted or 30 days older and in a severe autolysis state that compromised the histopathology examination were excluded from the study.

### 2.2 Necropsy

Pathology laboratories at veterinary hospitals usually use the organ block technique to perform necropsy in canine adults (Hataka et al. 2024). Another established technique involves a postmortem examination in a monoblock (Macpherson 1994, Stowell 1980, Valdes‐Dapena and Huff 1983, Waters 2009). Therefore, both techniques were combined to carry out a neonate necropsy.

**FIGURE 1:**
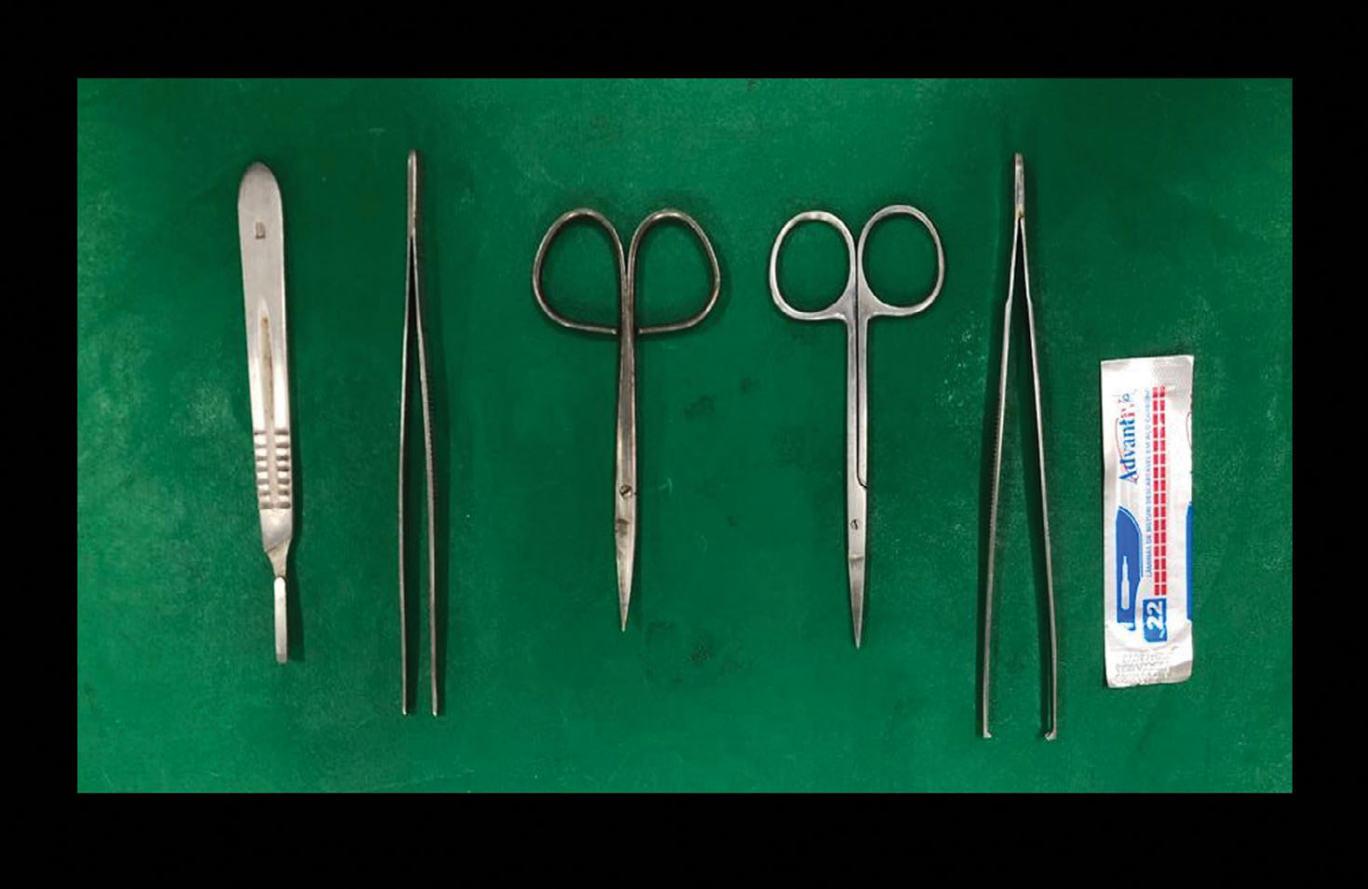
Materials for necroscopic examination, including (shown left to right in the photo): a scalpel handle for the initial incision of the corpse and removal of the monoblock and anatomical tweezer, from which the organs to be assessed will be separated; straight scissors for division; and a rat's tooth tweezer for exposure and collection of tissues for microscopic analysis.

The NIKON COOLPIX P900 camera, photo background and tool preparation were separated. The materials used for the necroscopic examination of neonates were extrapolated from the utensils used in necropsies of laboratory animals: scalpel handle, scalpel blade, straight and fine tip scissors, rat tooth dissection forceps and anatomical forceps (Figure 1).

**FIGURE 2:**
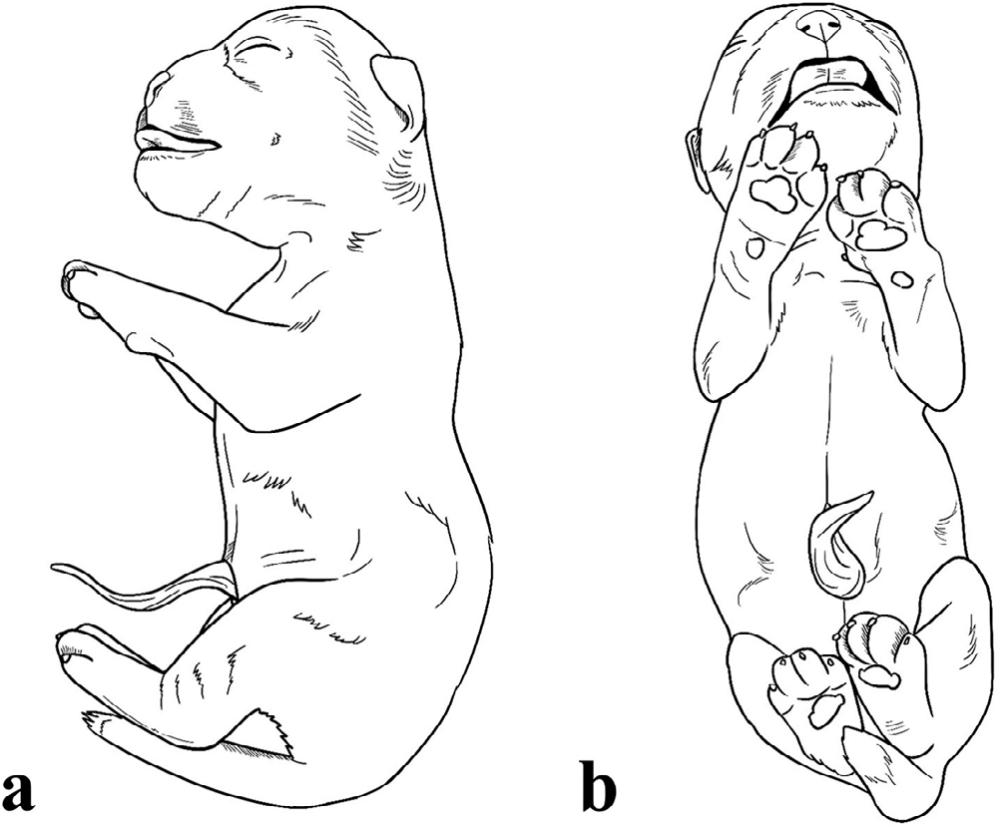
Schematic representation of the neonate. (a) Lateral view and (b) ventral view.

The complete clinical history of the canines involved in this study was obtained. Additionally, a requisition was provided containing the date, animal identification, species, sex, age, weight, breed, cause of death, time of death, duration of illness/antemortem diagnosis and information about the veterinarian in charge and the client, including their signature to authorise the procedure (Necropsy Examination Neonates—Supplementary material). Necroscopic examination was performed in steps as presented in Table 1 (Figure 2). Additionally, some tests are usually performed to detect abnormalities during necropsy (Hataka et al. 2024).

**TABLE 1:** Necroscopic examination stages in neonates.

Animal identification	Age Weight Size Breed Sex
External examination	Skin Eyes Nars Mouth Ear canal Anus Genitalia Congenital malformations
Internal examination	Subcutaneous Muscles and tendons Bones and joints Lymph nodes and thymus Blood and coagulation Cavities: oral, thoracic, abdominal and pelvic. Nervous system
Test	Negative pressure test (it allows the evaluation of the integrity of the thoracic chamber) Docimasia test (it shows if there was extrauterine life) Virchow's manoeuver (it checks the functioning of the bile flow)

The cadaver was positioned in the supine position and fixed with four needles in the distal joints of the limbs. The incision area was then moistened before the procedure began and a mentopubic incision was made via a scalpel.

**FIGURE 3:**
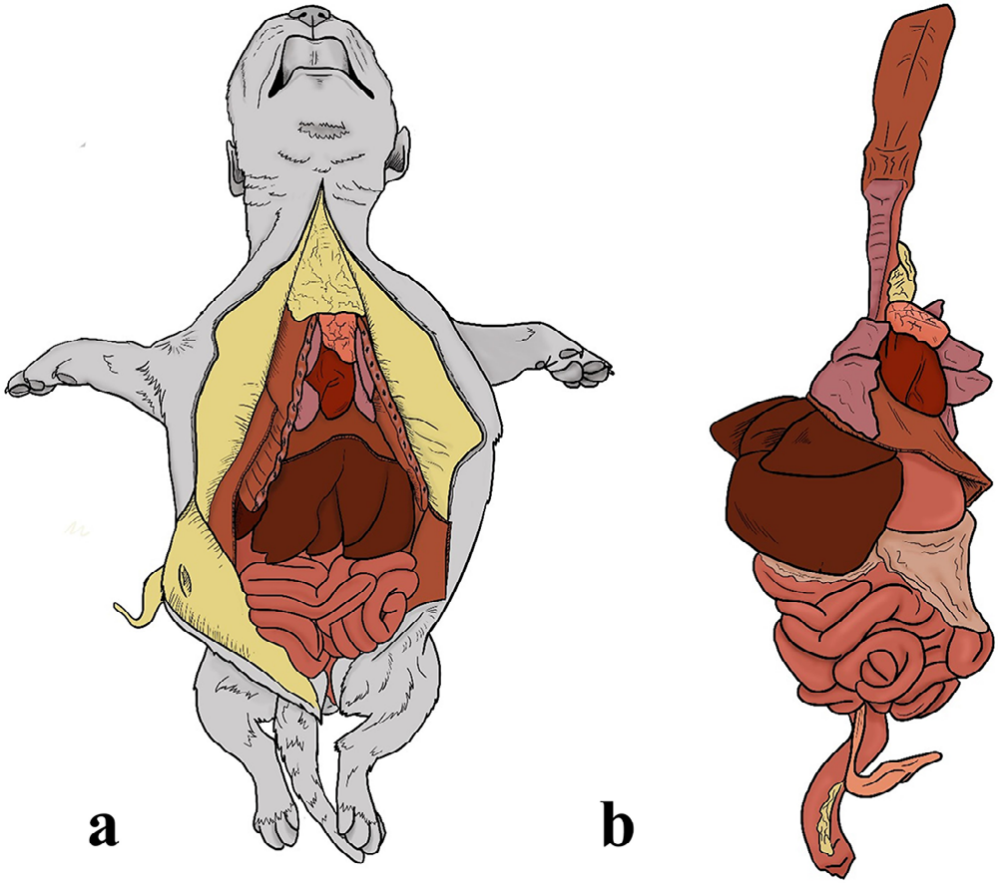
Schematic representation of the in situ evaluation of the neonate. (a) In situ evaluation of the cavity and (b) monoblock separation from the neonate body.

All the skin from the mentonian region to the pubis is pulled back laterally, as well as the thoracic musculature, including the forehead musculature, to exteriorise the costochondral joints. By using a scalpel in the craniocaudal direction from the midline to the pubis, a V‐shaped incision was subsequently made in the abdominal muscles, elevating the abdominal wall. Thus, the abdominal muscles were folded back and the organs were visualised (Figure 3).

The forelimbs and hindlimbs were disarticulated with the aid of a scalpel, with the hindlimbs disarticulated at the hip joint and the forelimbs folded laterally to fully expose the thorax (Figure 3).

The opening of the chest began with the assessment of negative pressure, which was performed by puncturing the diaphragm and observing its movement (the absence of wheezing indicates pneumothorax). This step was followed by the assessment of the pleural cavities by sectioning the costochondral joints with the costotome and removing the plastron. In addition to the plastron, the thymus, which is well developed in neonates and located near the base of the heart, can be observed.

The thoracic cavity was evaluated in situ to determine whether any free fluid was present in the cavity. Similarly, the organ volume and colour, degree of lung expansion or presence of topographical changes in the organs, mediastinal lymph nodes, and thymus were evaluated.

The in situ evaluation of the abdominal cavity allows assessment of the presence of free fluid in the cavity and measurement of volume, colour and gross appearance. The evaluation of the peritoneum can be performed considering its smoothness, brightness, transparency and presence of adhesions. Observation of organ topography can be performed, as can evaluation of the epiploon and evaluation of kidney position, symmetry and adhesions (in such cases, the right kidney can be identified prior to removal). Additionally, urinary bladder filling or voiding, together with the presence of adhesions, can be assessed (Figure 3).

The bloc was removed from all the organs and thoracoabdominal viscera. Two inverted ‘V’ incisions were made in the lateral branches of the mandible with a scalpel and the tongue was pulled in an anteroinferior direction with the index finger. The hyoid was subsequently disarticulated with a scalpel, followed by dissection of the cervical region muscles, loosening the trachea and oesophagus up to the entrance to the thoracic cavity, detaching the thoracic monoblock up to the diaphragm. An incision was made in the diaphragm in its posterior region and the monoblock was pulled caudally (Cardoso 2002, [Link]). The liver and stomach were released together and the large and small intestines were detached through an incision in the mesentery at a point at which the kidney was also removed. The pubis was sectioned and two incisions were laterally performed through the external genitalia and anus, thus freeing the entire monoblock (Nogué‐Navarro 2016, Orsini et al. 2019).

### 2.3 Monoblock Separation

The necropsy was conducted in six sequential stages to ensure a systematic and comprehensive examination of the anatomical structures.

- Respiratory and cardiovascular systems: The tongue, oropharynx, tonsils, trachea, oesophagus, lungs, heart and thymus were excised en bloc via a scalpel. The inferior vena cava was severed and the pulmonary ligaments attaching the lungs to the diaphragm were carefully incised.
- Lymphatic and digestive systems: The spleen and omentum were removed. Dissection of the omentum along the greater curvature of the stomach was performed via scissors, ensuring preservation of the pancreas, which is anatomically adjacent to the duodenum.
- Gastrointestinal tract: The final segments of the duodenum, jejunum, ileum, cecum and colon were excised. The section extending cranially to the urinary bladder was carefully isolated for further examination.
- Hepatobiliary system: The diaphragm was incised to facilitate removal of the liver, gallbladder, stomach, initial portion of the duodenum and pancreas.
- Genitourinary system: The adrenal glands were incised cranially, followed by systematic extraction of the remaining components of the genitourinary system.
- Musculoskeletal and nervous systems: If cerebrospinal fluid (CSF) collection was needed, it was performed prior to head removal via atlantooccipital disarticulation. A sagittal incision was made from the supraorbital region to the occiput to expose the skull. The cranial symphyses were then incised to detach the skullcap and access the brain. Brain extraction was performed in the rostrocaudal direction after the optic chiasm and cranial nerves were transected via scissors. Additionally, the joints were disarticulated for macroscopic evaluation of the synovial fluid, joint capsule and cartilage.

### 2.4 Morphological and Histological Analysis

In this study, samples were obtained from nine neonatal canines. The samples were conditioned in 10% buffered formalin and 70% ethanol at a 1:1 ratio for four days. Representative sections of the organs were systematically collected and fixed in neutral‐buffered 10% formalin during necropsy.

For histochemical analysis, tissue samples were fixed in 10% buffered formalin and embedded in paraffin. For haematoxylin and eosin staining, 4‐µM tissue sections were made via a semiautomatic microtome (Leica Biosystems, Wetzlar, Germany) and mounted on histological slides. Haematoxylin and eosin staining was performed following the laboratory protocol and the histological slides were evaluated under a light microscope (Leica Biosystems, Germany) by two coauthors (FBCM and NSR). Morphological evaluation was initially performed at low magnification (50x), followed by higher magnification (100x, 200x and 400x). The 50x magnification was used for tissue visualisation, allowing the identification of cellular components of different embryonic origins (mesodermal, ectodermal and endodermal). The presence of different specialised cell subtypes in each organ was analysed. The results are presented descriptively. A Masson's trichrome staining kit (Erviegas, Indaiatuba, Brazil) was used according to the manufacturer's instructions. Since there was no comparison group, this staining was evaluated qualitatively by describing the colour of the identified fibres (blue or red). The PAS technique was used to evaluate polysaccharide‐rich structures. PAS staining was performed via a commercial kit (Erviegas, Indaiatuba, Brazil) following the manufacturer's instructions. PAS evaluation was also performed qualitatively to assess the presence of polysaccharide‐rich vesicles in epithelial cells of the digestive tract.

Morphological analyses were performed on the neonates following the standards used in routine postmortem examination at the laboratory of the veterinary pathology laboratory in the veterinary hospital (Hataka et al. 2024). A qualitative analysis was conducted due to the small number of animals included in the study. The histopathology of the organs was described to determine their microscopic characteristics.

## 3 Results

Nine neonatal dogs were evaluated. Among them, four were males and five were females. The breeds included Yorkshire Terrier (3/9), Pug (1/9), French Bulldog (1/9), German Spitz (3/9) and mixedbred (1/9) breeds. The dogs weighed 120 to 300 grams and were, on average, 30 days old (early and late neonatal mortality) (Table 2). Following the time of death, the cadavers used in the study were evaluated within 2 h.

**TABLE 2:** General characteristics of necropsied canine neonates.

Animal	Sex	Race	Weight (g)	Age (days)	Size (cm)
1	Male	Yorkshire	150	0	7
2	Female	Mixed breed	150	0	7
3	Male	German spitz	300	15	10
4	Female	Pug	120	0	5
5	Female	French bulldog	170	0	7
6	Male	Yorkshire	160	3	6
7	Female	German Spitz	200	7	10
8	Male	Yorkshire	320	15	15
9	Female	German spitz	180	7	10

### 3.1 Gross Block Evaluation

**FIGURE 4:**
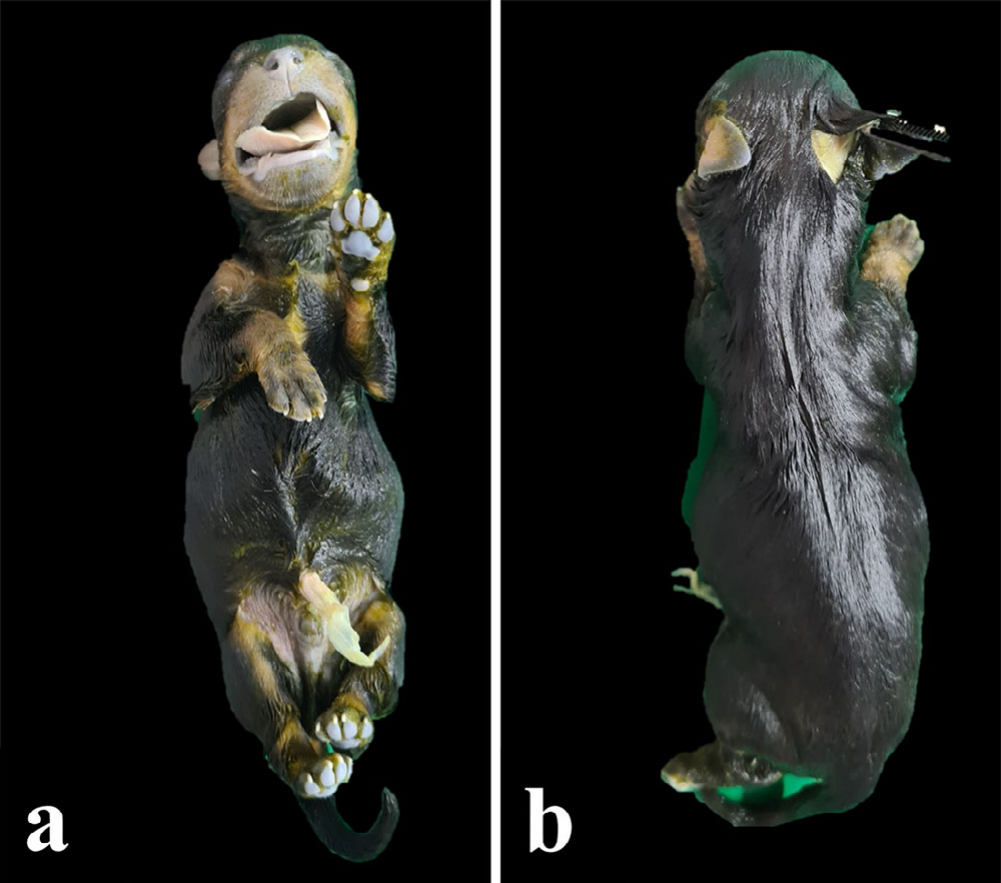
Gross external examination of the neonate. (a) Ventral view and (b) dorsal view.

Before the joint examination, the carcass, lymph nodes and muscles were assessed to identify possible haemorrhages, calcifications, exudates, parasites and other lesions. Similarly, the main joints, such as the patellar, carpal and tarsal joints, were assessed (Figure 4).

For the tongue, the external surface (size, colour, consistency and ulcers) was observed and sectioned longitudinally to make an internal assessment (colour, musculature and thickness of the mucosa). The oesophagus was opened continuously and passed through the trachea and carina with scissors and its contents were assessed (attention was given to the presence of foam, which may indicate pulmonary oedema). The oropharynx was cut, the mucosa (colour, changes, contents) was observed, and the tonsils were assessed.

The size, colour, consistency, changes and internal surface of the thyroid and parathyroid glands were assessed. The lung lobes were assessed both internally and externally. Notably, attention should be given to the presence of congestion and nodules; after external examination, transverse incisions were made in the pulmonary lobes. After the morphology and dilation of the heart chambers were observed, the organ was assessed via forceps and scissors through a small incision in the pericardial sac, from which the presence of fluid was observed. A longitudinal cut from the base to the apex of the heart was made to assess the presence of fluid, nodules or congestion. Additionally, the heart chambers and valves were also examined.

To assess the spleen, longitudinal sections were made using a scalpel. The colouration and adhesions of the omentum were also examined.

The intestinal loops were arranged in a zigzag pattern to assess their distension. Blunt scissors were then used to open the organ from the mesenteric border, followed by assessment of the contents (quantity, colour, consistency or parasites) and observation of the mucous membranes, including Peyer's patches and the presence of nodules or haemorrhages.

Virchow's manoeuver was performed before the stomach was separated from the liver. This manoeuver involves the application of digital pressure to the gallbladder to observe the outflow of bile through the duodenal papilla. The liver was separated from the diaphragm, and then the stomach was opened to assess its contents, performing the manoeuver in the direction of the cardia, greater curvature and pylorus.

The external and internal surfaces of the pancreas were evaluated through a longitudinal cut, the gallbladder and the diaphragm. The external and internal surfaces of the liver were evaluated via longitudinal sectioning.

The external and internal surfaces of the adrenals were assessed, the renal capsule was removed and a sagittal cut in the kidneys was made to assess the cortex, medulla and pelvis. The ureters and the external and internal surfaces of the urinary vesicle were evaluated. In females, the ovaries and the external surface of the uterus were assessed and the uterine horns were opened in a cranio‒caudal direction up to the vagina, which must also be assessed. In males, the prostate (inflammation, neoplastic masses), penis, foreskin and testicles were assessed.

The mucosa and contents of the rectum were evaluated. Urinary bladder opening via forceps and a scalpel is recommended. Owing to neonates' size and the fact that their reproductive systems are not yet fully developed, it is not always possible to assess the ovaries, uterus, uterine horns, prostate, penis, foreskin and testicles.

The cerebellar peduncles were sectioned, the cerebellum was removed and sectioning of the cerebellum was performed longitudinally. The thalamus and brainstem, as well as the telencephalon, were sectioned transversely. After fixation (20% formaldehyde), the brain was evaluated. For microbiological tests, sample collection must precede the fixation stage.

### 3.2 Main Alterations Observed During the Necroscopical Examination of Neonates

**FIGURE 5:**
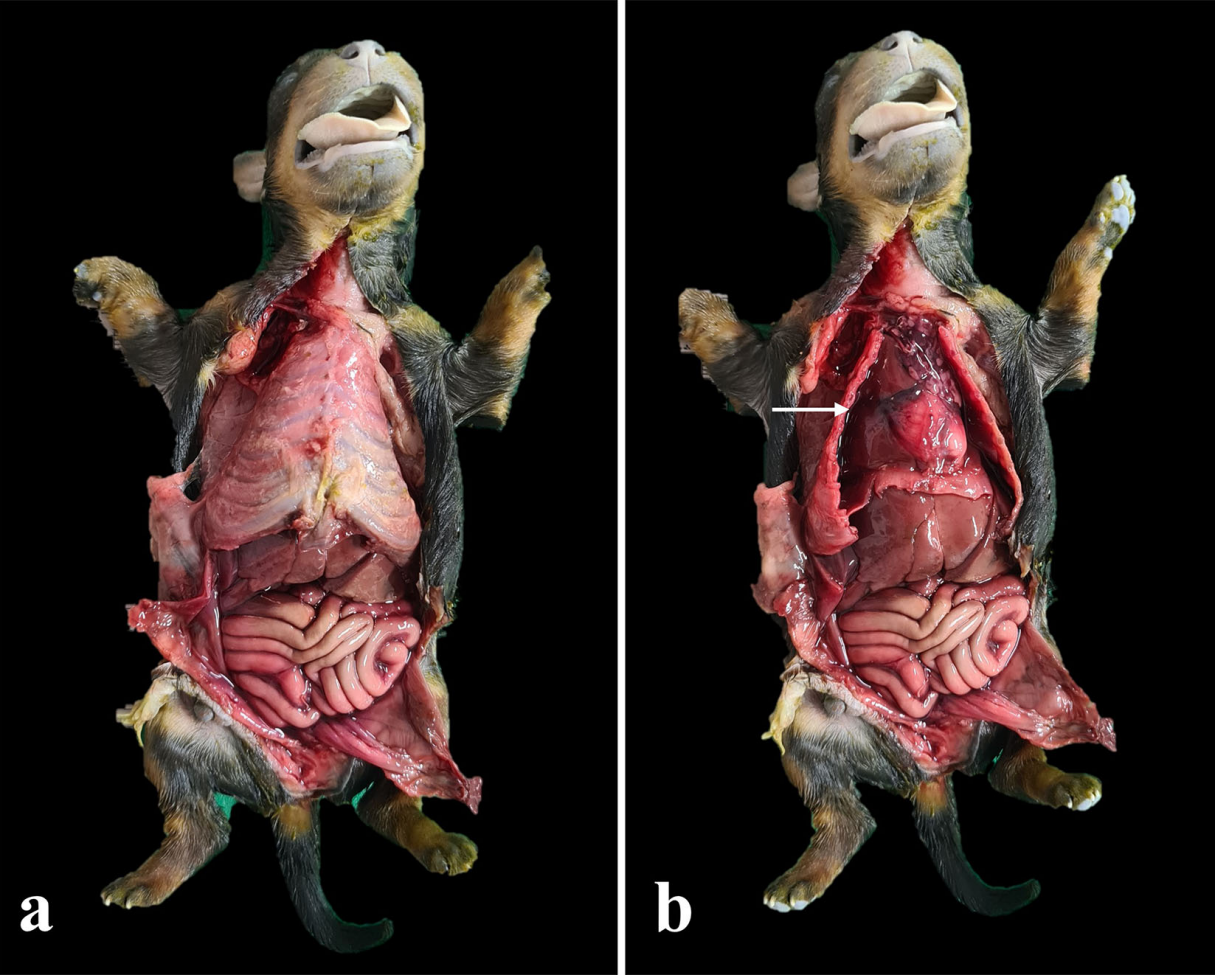
Internal examination of the neonate. (a) In situ evaluation of the cavity and (b) the hindlimbs disarticulated at the hip joint and the forelimbs folded laterally, exposing the thorax (white arrow).

**FIGURE 6:**
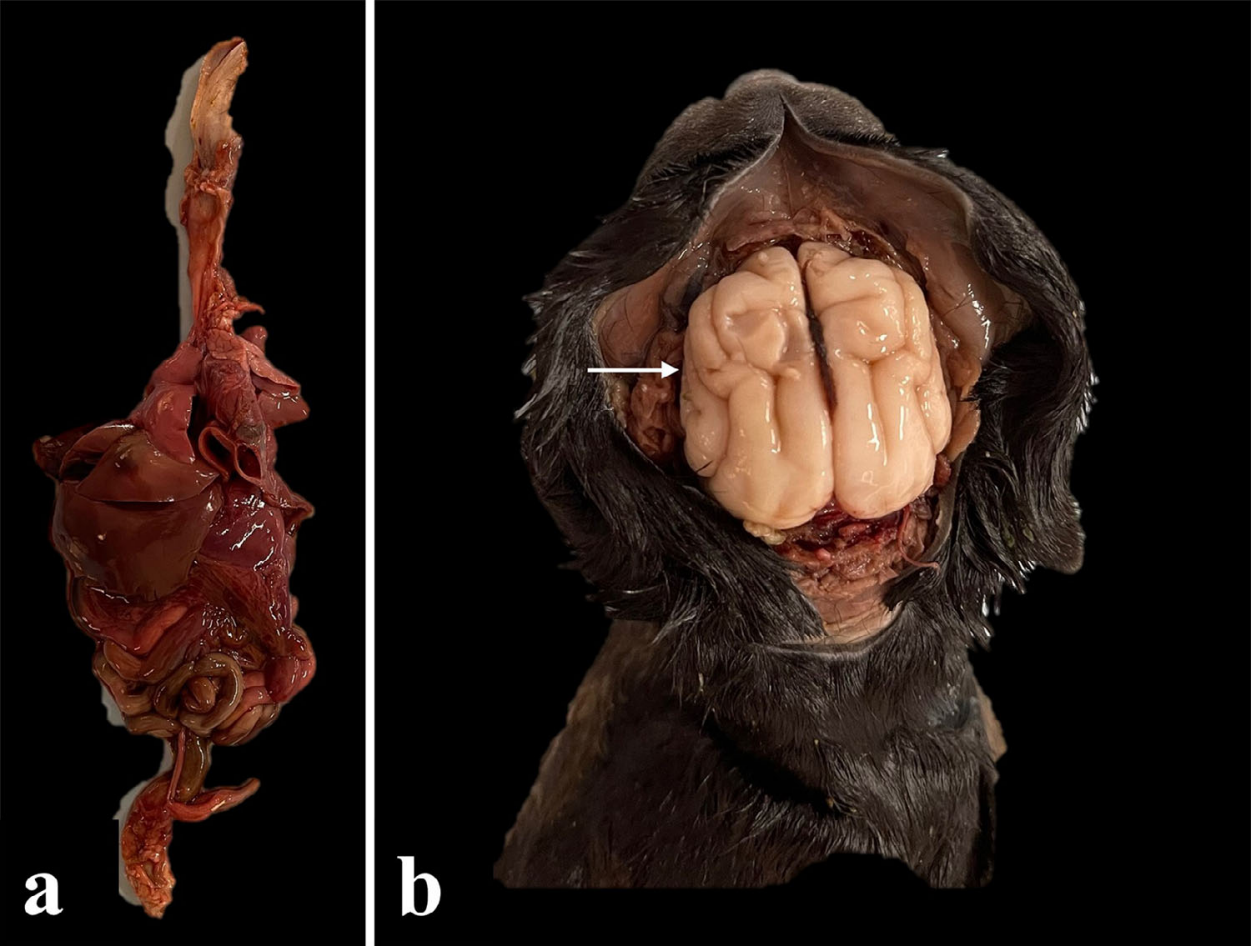
Gross image block removal. (a) Monoblock separation from the neonate body and (b) skull cap removal exposes the nervous system (white arrow).

**FIGURE 7:**
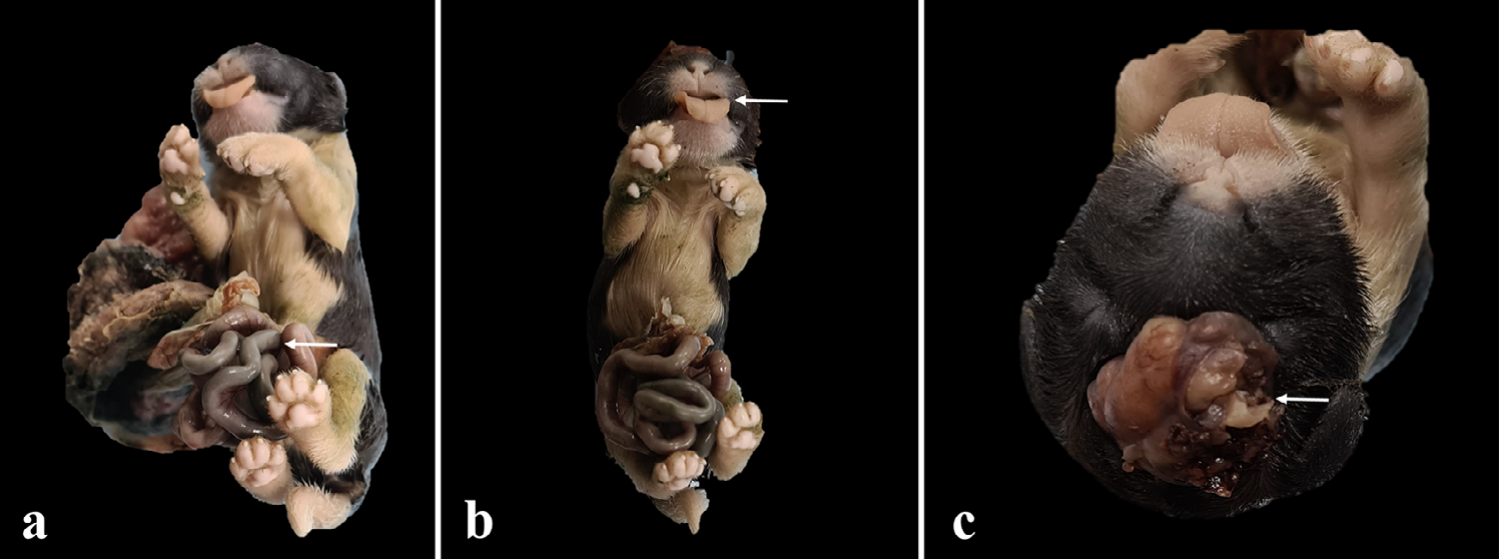
Gross image of congenital malformations. (a) Omphalocele (white arrow), (b). macroglossia (white arrow) and (c) encephalocele (white arrow).

Some alterations were observed during the macroscopic examination of neonate cadavers. First, some dogs presented autolysis (Figures 5 and 6) (Table 3). Additionally, one case of congenital malformation revealed omphalocele, macroglossia and encephalocele (Figure 7).

**TABLE 3:** Macroscopic changes observed at necropsy.

Change	X	%
Moderate to severe autolysis	3	33
Congenital malformations	1	11
Presence of foamy content in the trachea (suspected pulmonary oedema)	4	44
Visceral increase	1	11
Presence of abnormal gastric or intestinal content	2	22
Enlargement of lymph nodes	0	0
Visceral haemorrhages	1	11

### 3.3 Gross Histopathology

The histological evaluation revealed that the mucosa layer of the tongue runs from the top to the base and presents keratinised stratified squamous epithelium (Figure 7) (Table 4). The lamina propria contains loose connective tissue. The submucosa is composed of connective tissue, salivary glands, skeletal muscle and salivary ducts (Figure 7). The mucosa of the stomach presented a simple columnar epithelium specialised in mucus secretion, a lamina propria with loose connective tissue and immune cells and an underdeveloped muscular mucosa. The *muscularis propria*, with inner circular and outer longitudinal layers of smooth muscle, was thinner and less differentiated. The stomach serosa was thin and had few collagen fibres and the parietal cells and gastric glands were less developed (Figure 7). The intestines contain a mucosa containing enterocytes with microvilli to increase the absorptive surface area and goblet cells that secrete mucus. The lamina propria presented loose connective tissue with blood vessels, lymphatics and a thin layer of smooth muscle. The submucosa consists of dense connective tissue and the muscular externa presents with two layers of smooth muscle (inner circular and outer longitudinal) that are responsible for peristalsis. The serosa, composed of connective tissue and covered by mesothelium, was observed (Figure 7). The duodenum is characterised by a mucosa with elongated, thin villi covered by a simple columnar epithelium consisting of absorptive cells (enterocytes) and mucus‐secreting goblet cells. The submucosa houses Brunner's glands, which secrete alkaline mucus to help neutralise gastric acid. Similarly, the muscularis layer, which consists of an inner circular layer and an outer longitudinal layer facilitating peristalsis, was noted.

**TABLE 4:** Histopathological findings by organ.

Organ	Histological changes observed	Frequency (n/9)	%
Tongue	Underdeveloped keratinised squamous epithelium	9	100
Stomach	Poorly developed gastric glands	5	55
Intestine	Immature villainy, shallow crypts	8	88
Liver	Small hepatocytes, broad sinusoids	9	100
Pancreas	Few and immature Langerhans islets	9	100
Lung	Immature cartilage, little alveolar maturation	4	44
Heart	Immature cardiomyocytes, irregular organisation	4	44
Rim	Immature renal corpuscles in the cortical region	5	55
Bladder	Fine urothelium, underdeveloped musculature	9	9
Spleen	Poorly defined white pulp	9	9

**FIGURE 8:**
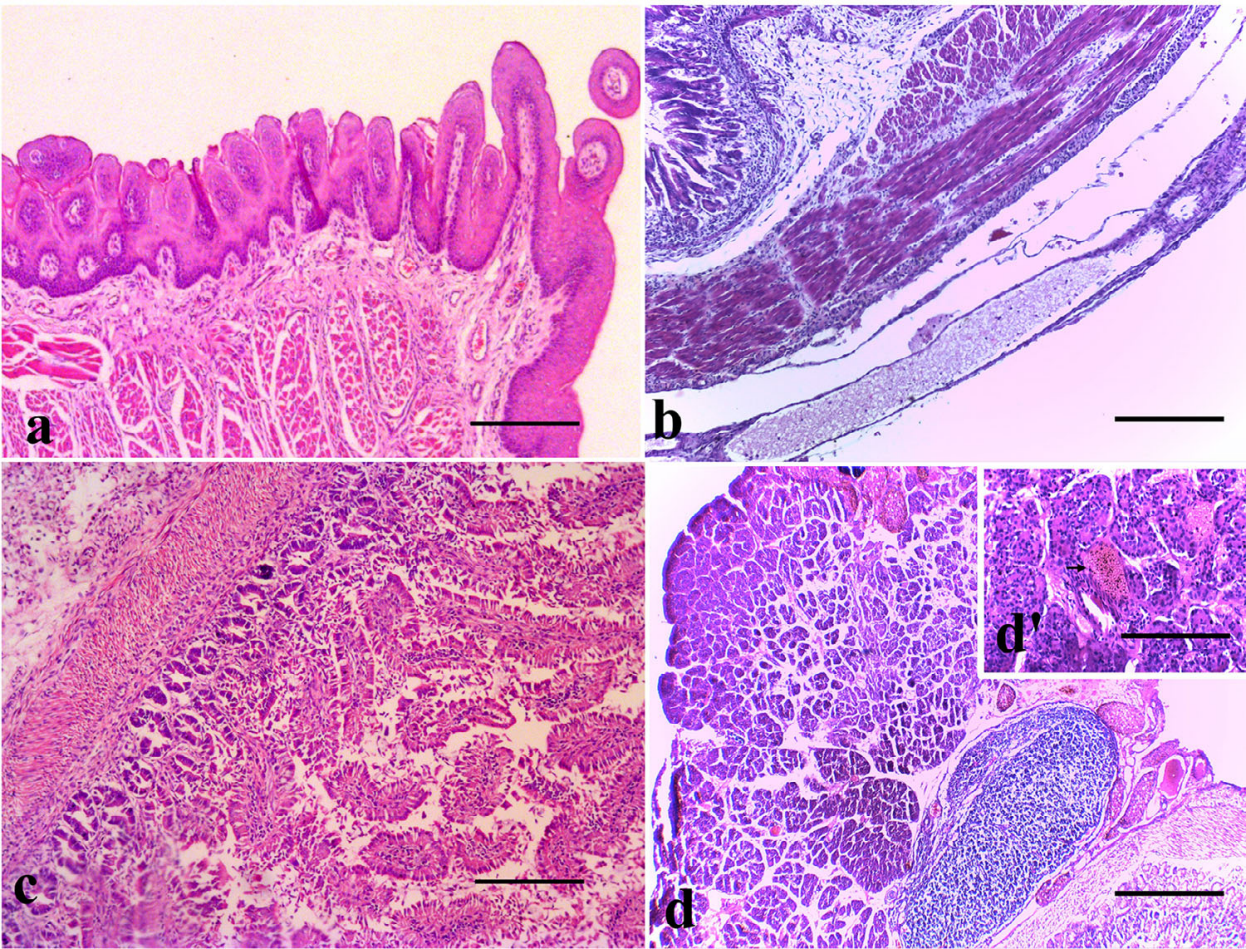
Gross histopathology of the digestive tract. (a) Tongue layers presenting keratinized stratified squamous epithelium in the mucosa followed by the lamina propria and the skeletal muscle in the submucosa, (b) stomach layers showing the inner circular and outer longitudinal layers of smooth muscle and the serosa with collagen fibers. (H&E, bar = 200 µm), (c) intestine layers subjected to autolysis (H&E, bar = 100 µm) and (d) gross architecture of the pancreas (H&E, bar = 200 µm) d’. The pancreas parenchyma contains the islets of Langerhans (black arrow) (H&E, bar = 50 µm).

Following histopathological evaluation, the liver parenchyma consisted of large polygonal cells called hepatocytes, which were smaller and less organised in neonates and contained more basophilic cytoplasm. In addition, we observed that the hepatic sinusoids and portal tracts were less developed and more spaced out in the hepatic parenchyma of neonates. The spaces of Disse, gaps between hepatocytes and the sinusoidal endothelium, were also wider in neonates because of the lower density of hepatocytes. The pancreas parenchyma was shown to have islets of Langerhans that are less developed and fewer in number than those seen in adults, with beta cells and alpha cells still maturing in the neonate. The exocrine component consists of pancreatic acini, which are clusters of acinar cells responsible for the production of digestive enzymes. These acini appeared to be less developed and less densely packed in neonates, with a basophilic cytoplasm. The pancreatic ducts, including the intercalated and intralobular ducts, are less organised and more immature in neonates. The stroma of the pancreas, which is composed of connective tissue, contains blood vessels and nerves; however, it is less dense and more loose in neonates (Figure 8).

**FIGURE 9:**
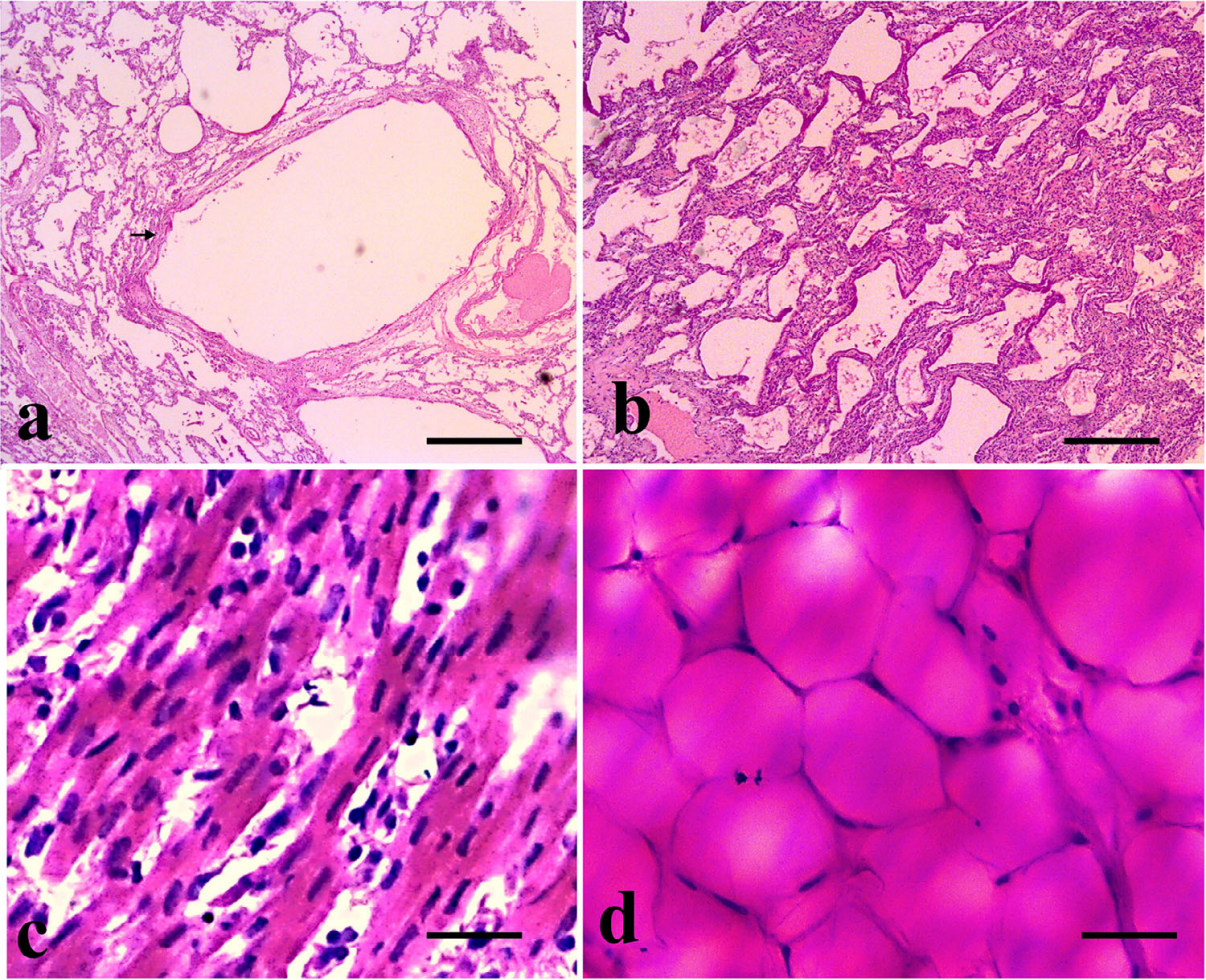
Gross histopathology of the lungs and heart. (a)–(b) Developmental bronchi (black arrow) presenting immature cartilage (H&E, bar = 100 µm) and (c)–(d) cardiac striated muscle with longitudinal and transverse filaments (H&E, bar = 50 µm).

The lung parenchyma consists of primary bronchioles with simple columnar respiratory epithelium, smooth muscle, bronchial hyaline cartilage, terminal bronchioles with simple cuboidal epithelium, alveolar ducts, alveolar sacs and alveoli. The development of the bronchi presented immature cartilage, which was initially eosinophilic due to the absence of proteoglycans (Figure 9). The heart, an organ divided into four chambers (atria and ventricles) and separated by valves, was evaluated, with the cardiac parenchyma consisting of an epicardium supported by a layer of dense irregular connective tissue and loose adipose connective tissue. In the myocardium, cardiomyocytes with contractile filaments called sarcomeres that emerge in the striated muscle of the heart were observed (Figure 9).

**FIGURE 10:**
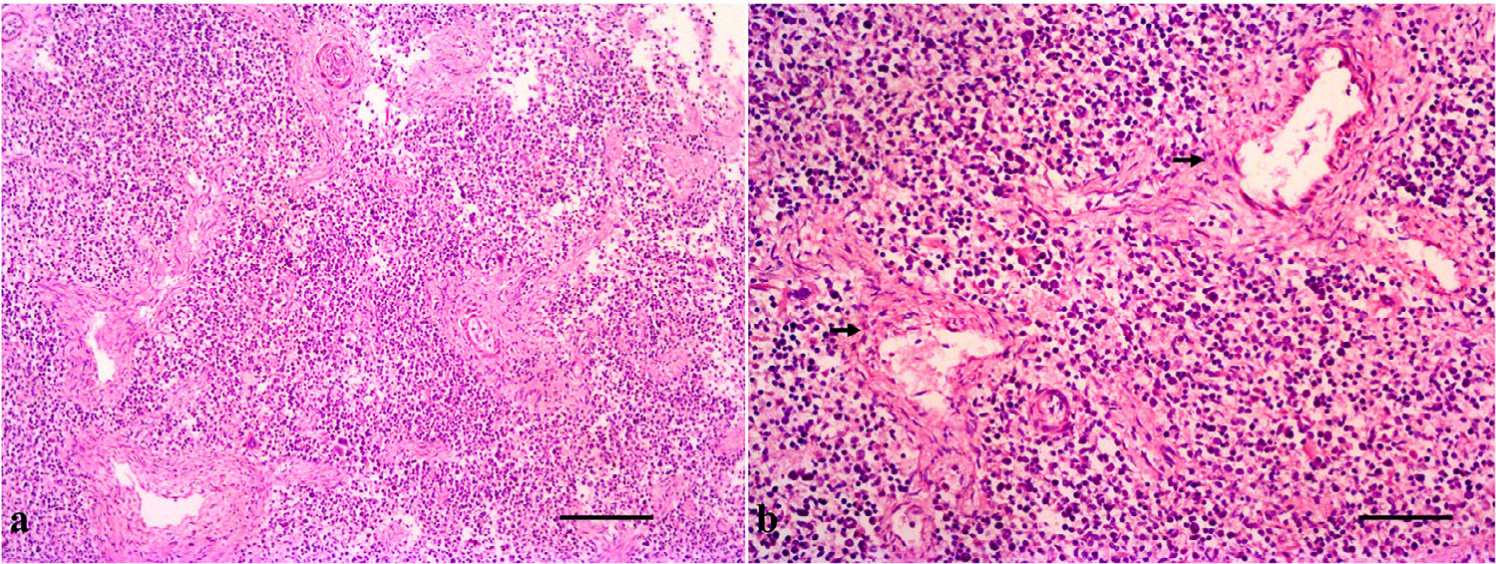
Gross histopathology of the spleen. (a) Gross architecture of the spleen (H&E, bar = 200 µm) and (b) thin fibrous capsule extending internally and forming trabeculae (black arrow) (H&E, bar = 100 µm).

The spleen has a thin fibrous capsule that extends internally, forming trabeculae that provide structural support and conduct blood vessels. The white pulp mainly consists of lymphocytes around the central arteries, with developing lymphoid follicles. The red pulp was the predominant sinusoid and the splenic cords represented its main composition. The spleen was richly vascularised, with arteries entering through the capsule and branching into trabeculae, forming central arteries in the white pulp and sinusoids in the red pulp (Figure 10).

**FIGURE 11:**
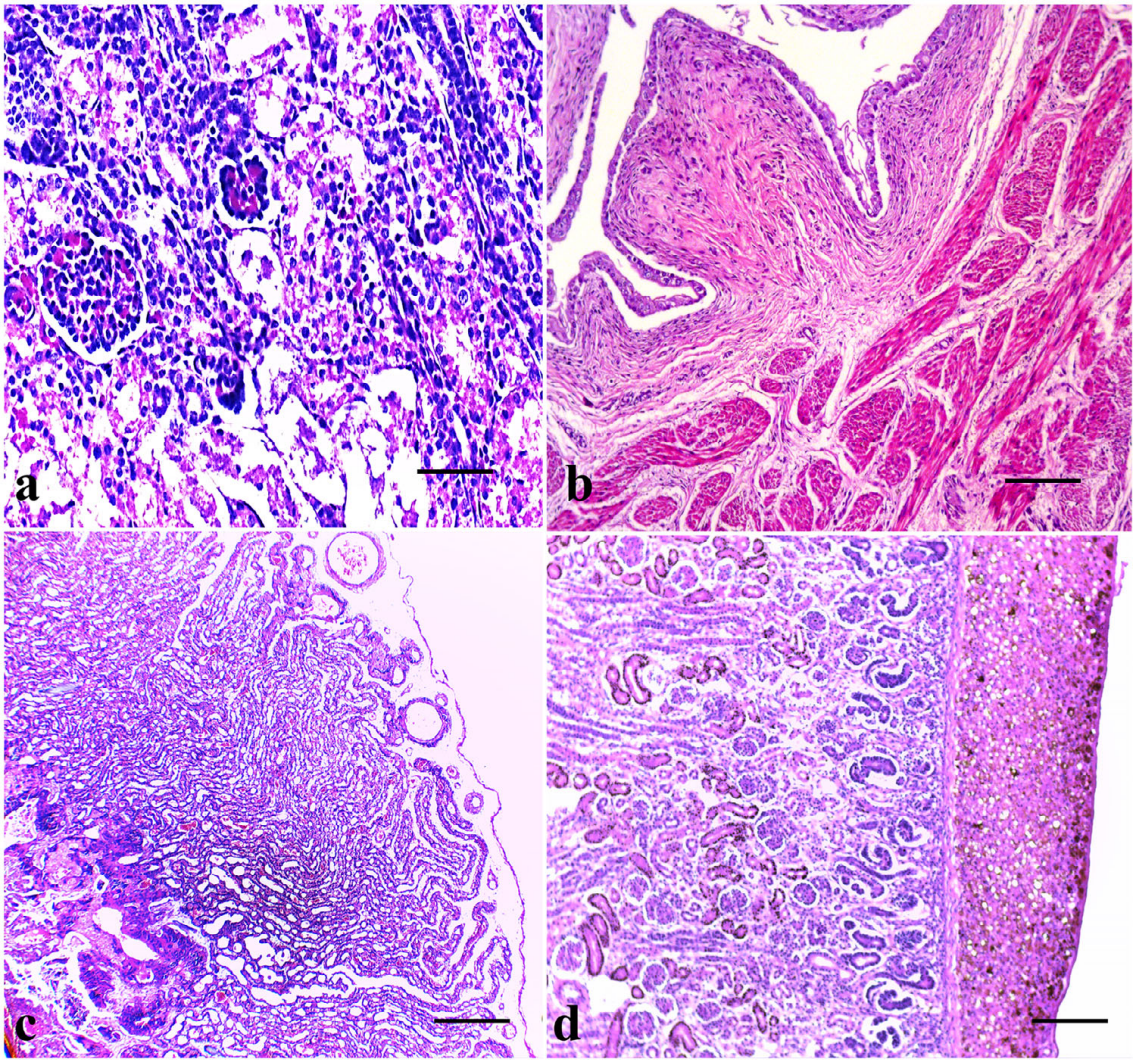
Gross histopathology of the kidney, bladder and placenta. (a) Developing corpuscles in the outer cortex at various stages of completion. (H&E, bar = 50 µm), (b) the bladder has four layers: the urothelium, the lamina propria and the *muscularis externa* (H&E, bar = 100 µm), (c) the placental arteries (black asterisk) surrounding the placenta parenchyma are visible (H&E, bar = 200 µm) and (d) the stratum basalis in which the villi are anchored (H&E, bar = 100 µm).

**FIGURE 12:**
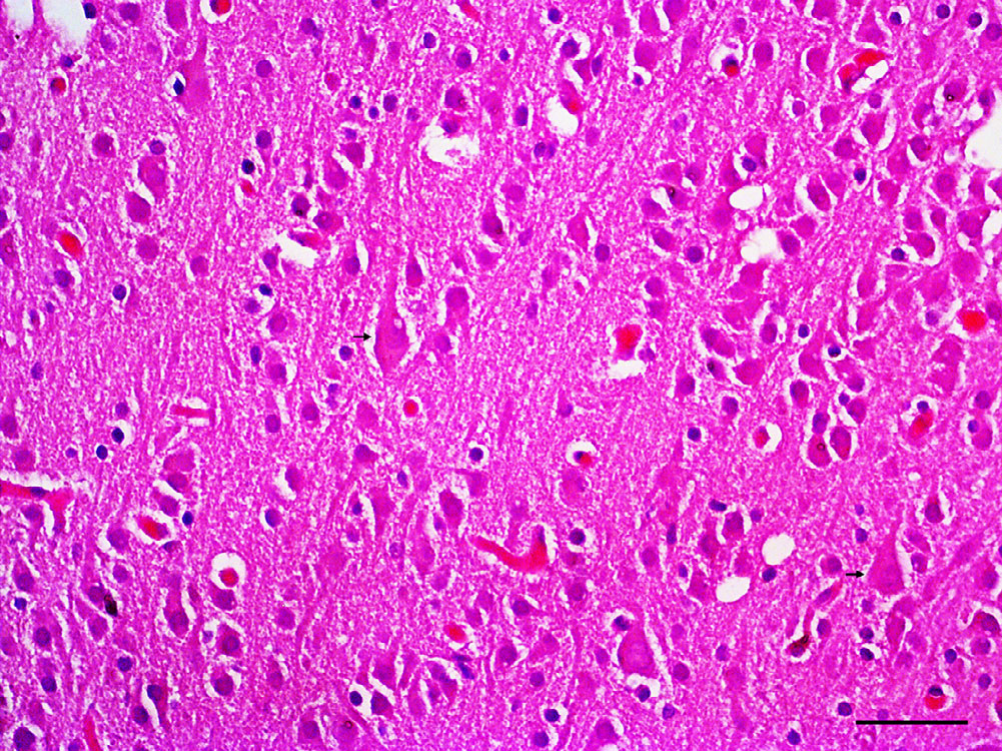
Gross histopathology of the brain. Brain with autolysis showing neurons (black arrow) of different sizes and shapes (H&E, bar = 50 µm).

The kidneys are divided into cortical and medullary portions; in neonates, it is possible to differentiate mature corpuscles in the inner cortex and developing corpuscles in the outer cortex in various stages of completion. As expected, the renal corpuscles contained the Bowman capsule and the glomerulus, which are involved in the cortical labyrinths containing the eosinophilic proximal and distal convoluted tubules (Figure 11). The lighter inner region is the medulla, in which it is possible to observe the collecting ducts, the proximal and distal straight tubules and Henle's loop. The urinary bladder has four layers: the urothelium, the lamina propria and the muscularis externa. The smooth muscles in the two layers in the muscularis externa were loosely arranged. The lamina propria consisted of a prominent dense irregular connective tissue layer. The urothelium contained the transitional epithelium (Figure 11). Moreover, the placenta presents the stratum basalis in which the villi are anchored (villi are projections of the foetal chorion that extend into lacunae through which the maternal blood flows). However, placental artery development from the endometrial arteries to supply maternal blood to the lacunae was observed (Figure 11). The brain comprises the central nervous system, in which microscopic analysis revealed neurones that varied in size and morphology (Figure 12).

## 4 Discussion

Necropsy is a valuable diagnostic tool for identifying the causes of neonatal mortality in dogs. Despite its importance, necropsies of neonatal animals remain underutilised by veterinarians (Coelho 2002, Mila et al. 2021). This study highlights the necessary steps for neonatal postmortem examinations and provides an anatomical and histological characterisation of neonatal organs, offering insights into the challenges associated with this procedure.

Performing necropsies on neonates is challenging due to their small size and distinct anatomical and physiological characteristics. Congenital malformations, for example, can significantly impact neonatal viability (Agnew 2023). In this study, one puppy presented with omphalocele, macroglossia and encephalocele, conditions identified during macroscopic examination, which likely contributed to its mortality. Similarly, infections such as canine herpesvirus (CHV) are known to cause abortion, stillbirth and neonatal death, with haemorrhagic characteristics in the lungs, liver and meninges, as well as intranuclear inclusion bodies and necrosis observed microscopically (Junqueira and Carneiro 2017). However, no evidence of this viral infection was found in the puppies analysed.

The establishment of a standardised neonatal necropsy protocol is crucial for determining causes of death, identifying disease patterns, and improving neonatal health. A structured examination allows for better documentation of pathological alterations, facilitates research comparisons and enhances diagnostic accuracy (Coelho 2002). Postmortem evaluation is suggested to begin with a thorough external inspection, assessing ectoparasites, mucous membrane colouration, alopecia, skin wounds, fractures and other physical abnormalities. The animal was positioned in the dorsal decubitus position for examination. Cadaveric changes can be observed as early as 30 min postmortem (Hataka et al. 2024, Lamm and Njaa 2012).

For organ removal, the monoblock technique, which extracts organs as a single unit, was employed, considering the anatomical and physiological differences between neonates and adults (Stowell 1980, Waters 2009). Histopathological analysis revealed significant structural differences in neonatal tissues compared with adult tissues. For example, gastric glands and parietal cells are less developed, reflecting ongoing maturation, whereas hepatocytes exhibit a more basophilic cytoplasm, indicative of active protein synthesis, similar to observations in human neonates (Samuelson 2007). Additionally, organs such as the pancreas, lungs, kidneys and spleen display developmental characteristics, emphasising their role in metabolic adaptation during the early stage of life (Deleberre 2014).

This study underscores the importance of standardising neonatal necropsy protocols to improve diagnostic precision and support veterinarians in interpreting findings. A systematic approach to postmortem examinations can contribute to reducing neonatal mortality and improving both individual and collective neonatal health.

## Author Contributions

**Victoria Ghedin, Jéssica Cardia de Melo, Fernanda Barthelson Carvalho de Moura, Keylla Helena Pereira, Fernando Carmona Dinau, Pedro Ximenes, Natália Freitas de Souza, Maria Lucia Gomes Lourenço, Sérgio Felisbino, Tatiane T Negrão Watanabe and Noeme Sousa Rocha**: Conceptualisation, visualisation. **Victoria Ghedin, Jéssica Cardia de Melo, Fernanda Barthelson Carvalho de Moura, Carlos Mario Gonzalez Zambrano, Juliana Jurado Jiménez, Gabriela Abreu Botelho, Luiz Guilherme Dercore Benevenuto, Reiner Silveira de Moraes and Ana Júlia Motta da Costa**: Writing—original draft. **Keylla Helena Pereira, Fernando Carmona Dinau, Pedro Ximenes, Natália Freitas de Souza, Maria Clara Boni Raffi, Tatiana Pessoa Onuma, Maria Lucia Gomes Lourenço, Sérgio Felisbino, Tatiane T Negrão Watanabe, Monica Barthelson Carvalho de Moura, Luis Mauricio Montoya‐Flórez, Francisco Pedraza‐Ordoñez and Noeme Sousa Rocha**: Writing—review and editing. **Noeme Sousa Rocha**: Supervision.

## Ethics Statement

This study was approved by the Ethics Committee on Using Animals in Research (CEUA‐ #0031/2021) of São Paulo State University (UNESP), School of Veterinary Medicine and Animal Science, Botucatu.

## Supporting information



Supporting information

## Data Availability

Simulation files and other data can be obtained from the corresponding author upon reasonable request.
