# Supplementary material for: Necropsy Technique and Histological Characterisation of Organs From Neonatal Puppies: What Do We Know?
Source: Vet Med Sci. 2025 May 13;11(3):e70392. doi: 10.1002/vms3.70392 (PMC12075929; doi:10.1002/vms3.70392)
Supplement: Supplementary file 1 — Supporting information [file VMS3-11-e70392-s001.docx]

**NECROPSY EXAMINATION NEONATES**

**Identification of the animal:**

| Age: | Peso (Kg): | Size (cm) Size |
| --- | --- | --- |
| Race: | Sex: |  |

**A. External examination: External examination**

1.Skin (hydration, skin lesions, ectoparasites): ______________________________________________________________________________________________________________________________________________

2. Eyes (filling of the eye sockets, appearance of the cornea, myrosis or myosis, opacity or transparency of the lens, ocular secretions): ______________________________________________________________________________________________________________________________________________

3. Nose: ________________________________________________

4. Mouth (mucosa oral, changes in the gum or tongue, teeth, cleft palate/palate):___________________________________________________________________________________________________________________________

5. Ear Canal (injury and discharge):______________________________________________________________

6. Anus (injury and discharge):_________________________________________________

7. Genitalia (penile or vulvar injury and secretion):_______________________________

8. Congenital malformations (anasarca, palatosquise, gastroquis, onfalocele ...): _______________________________________________________________________

**B. Internal examination**

1. Subcutaneous:_________________________________________________________

2. Musculature and tendons: _______________________________________________

3. Bone and joints: _______________________________________________________

4. Lymph nodes: _________________________________________________________

5. Blood and coagulation (Thromb):__________________________________________

**B1. Oral and Neck Cavity:**

Pharynx, larynx, trachea, esophagus, thyroid, parathyroid, salivary glands and lymph nodes:_________________________________________________________________________________________________________________________________________________

**B2. Thoracic cavity:**

In situ examination (free liquid - volume, color, appearance; change in the topography or size of organs; neoplasms; pleura-adhesions, alterations; and lung - staining, adhesions; heart size): Size:__________________________________________________________________________________________________________________________________________________

1.1 Negative pressure test:

Present: Absent:

2. Lung, heart, pleura, pericardial sac, lymph nodes:_____________________________________________________________________________________________________________________________________________________

2.1 Docimasia test:

Positive: Negative:

**B3. Abdominal and Pelvic Cavity:**

1. In situ examination (free liquid - volume, color, aspect; change in the topography or size of organs; neoplasms):________________________________________________________________________________________________________________________________________________

2. Spleen and epiplon:________________________________________________________

Intestine, mesentery and mesenteric lymph nodes:__________________________________________________________________

4. Diaphragm, liver, gallbladder, stomach, duodenum and pancreas:_____________________________________________________________________

4.1 Virchow’s maneuver:

Positive: Negative:

5. Adrenal, kidney, urethra, bladder, urethra, rectum, anus, aorta/caudal cavity, ovaries, uterus, vagina, vulva, prostate, penis and testicle:________________________________________________________________________________________________________________________________________________________________________________________________________________________________

**B.4 Nervous system**

1. Brain and Bone Marrow:_____________________________________________________

- **Checklist of samples for histopathology:**

**Lung**

**Liver**

**Heart**

**Kidneys**

**Adrenals**

**S Estomach**

**Small Intestine (Duodenum, Jejunum and Ileum)**

**Large Intestine (Cecum, Colon and Rectum)**

**Spleen**

**Thyroid**

**Timo**

**Pancreas**

**Lymph nodes**

**Salivary glands**

**Encephalon**

**Tongue**

**Ovaries**

**Uterus**

**Bladder**

**Testicle**

**Prostate**

**Other(s) fabric(s):_______________**

**
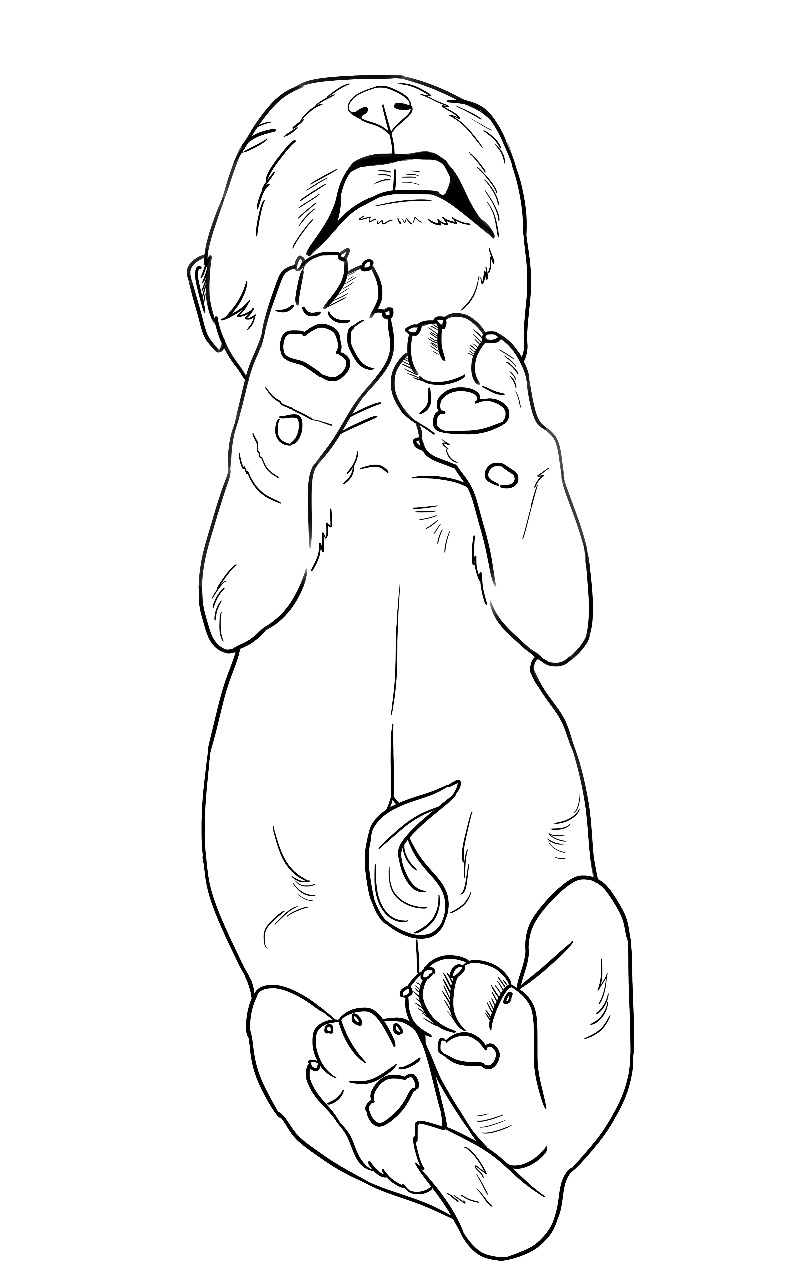

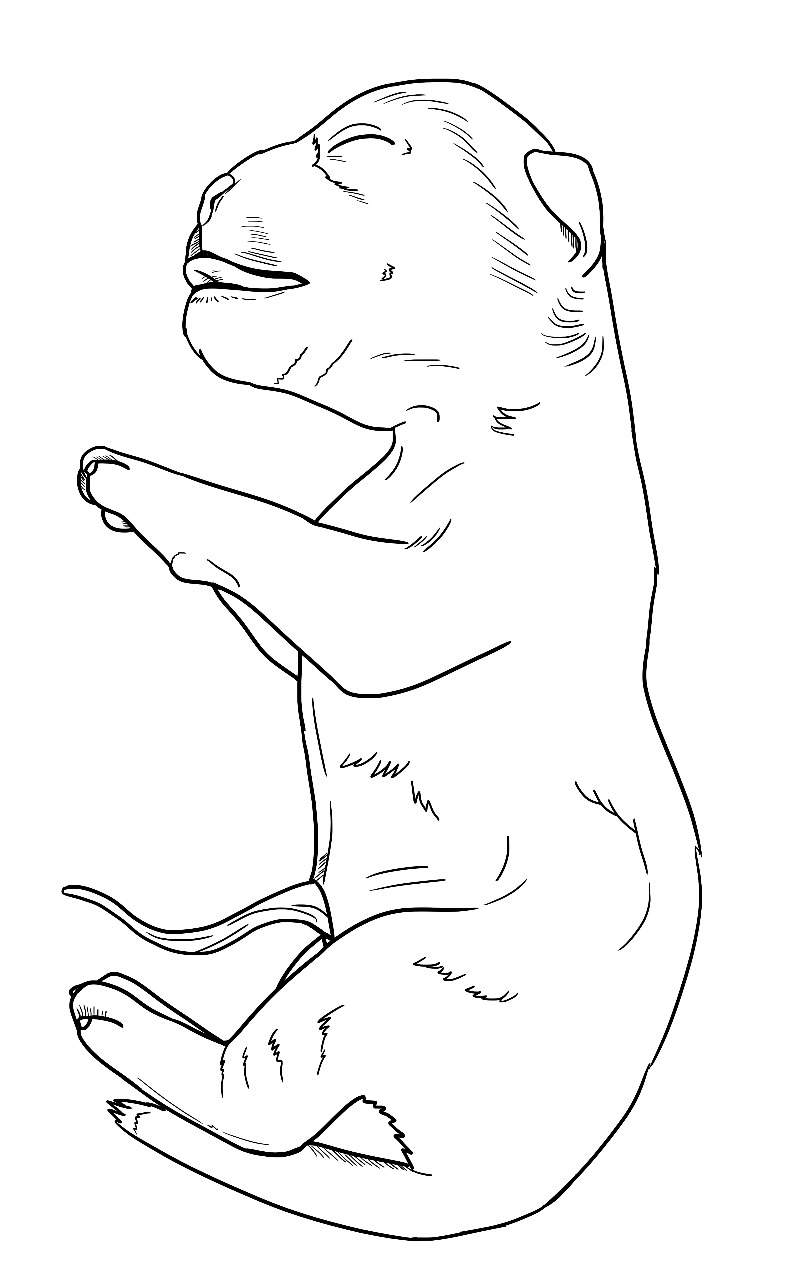
**

**
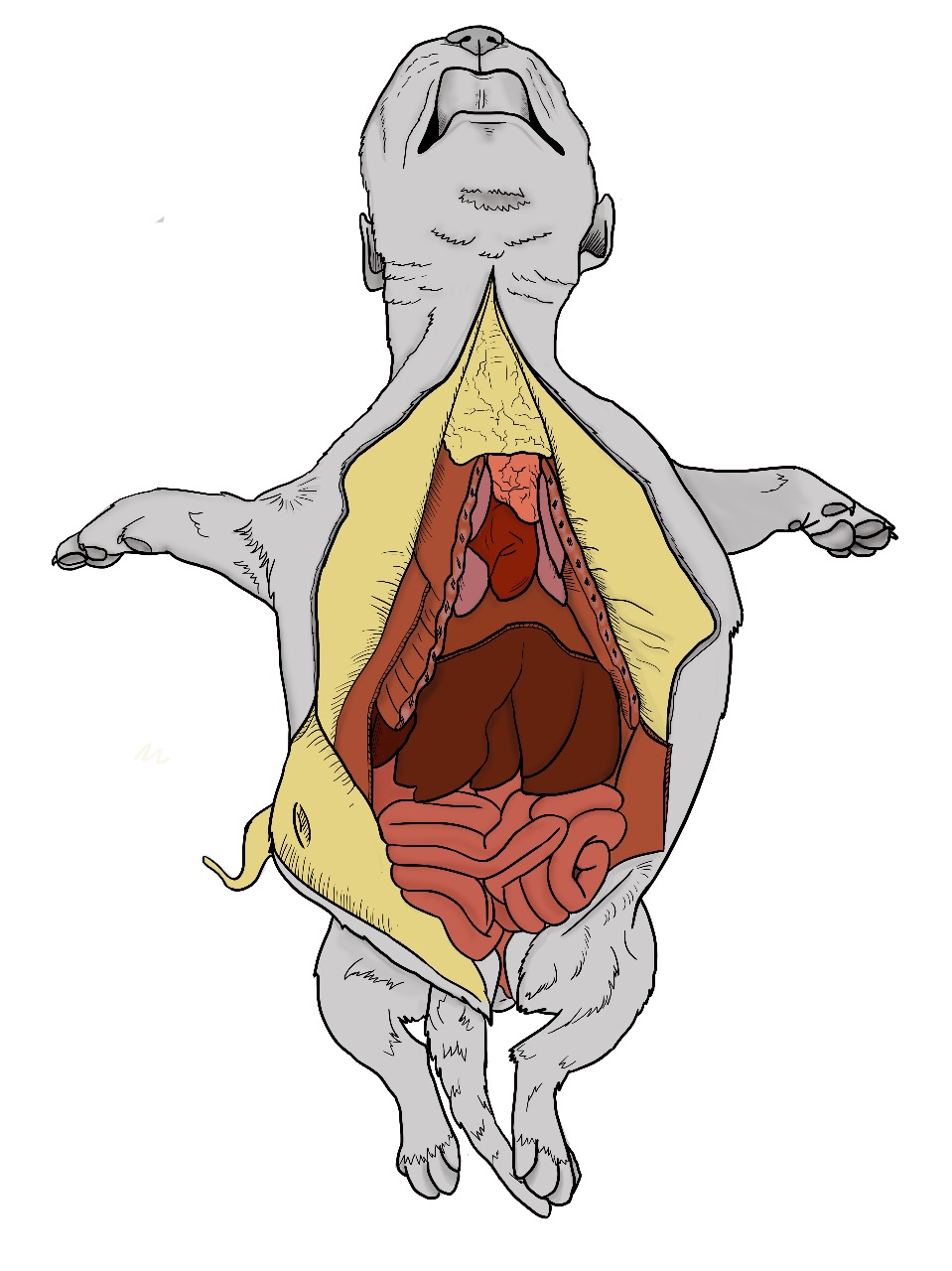
**

**
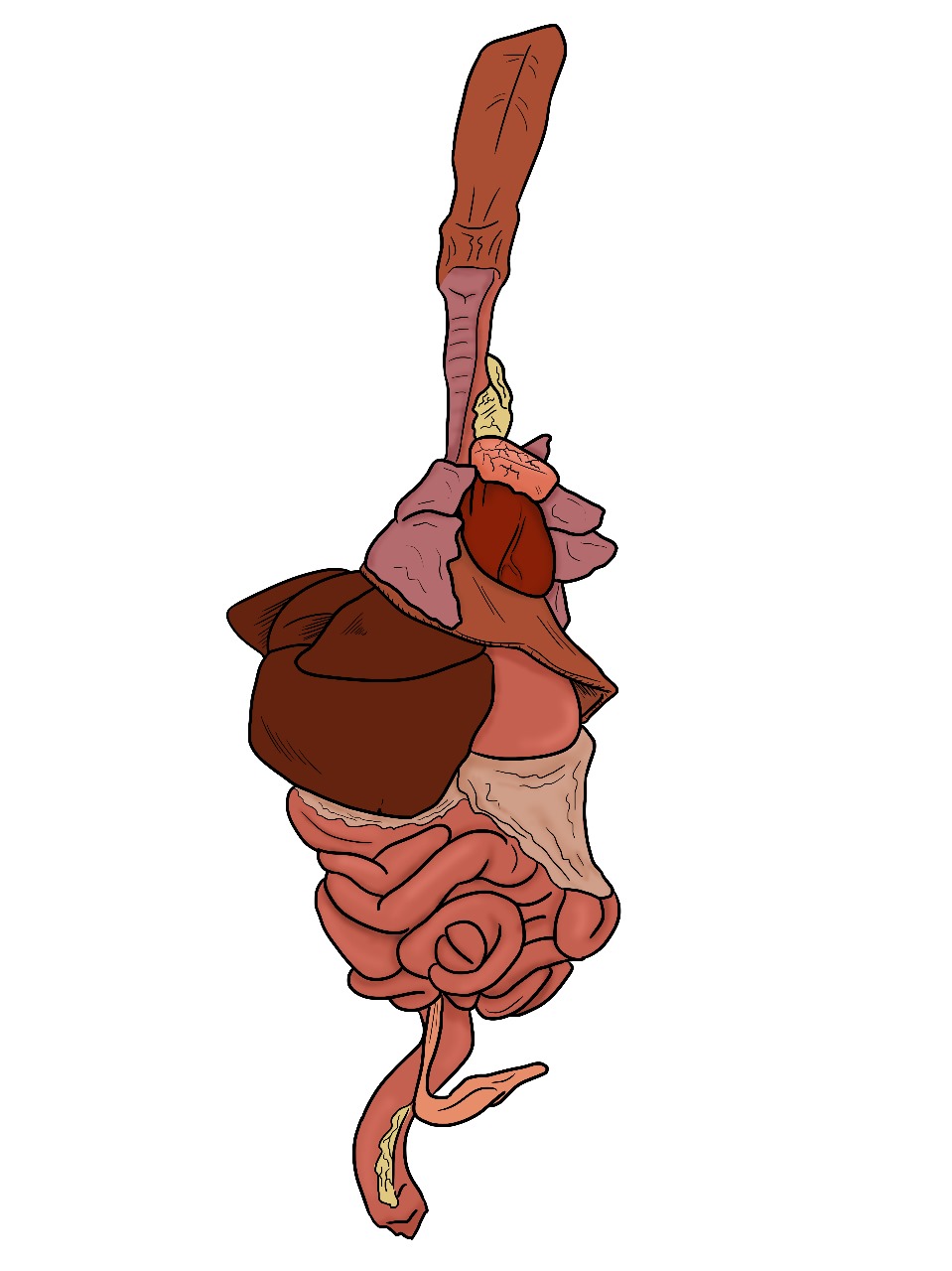
**

**
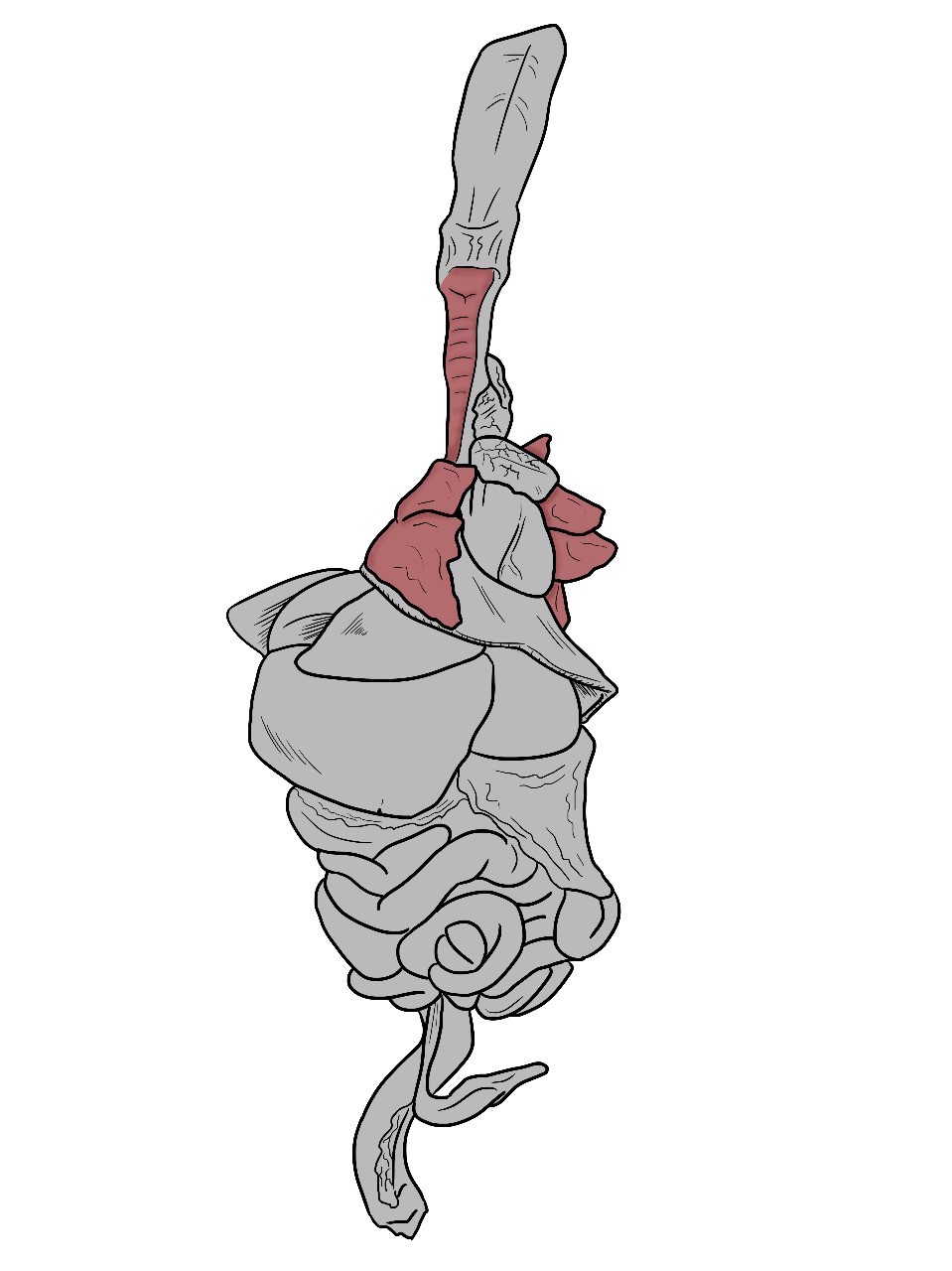

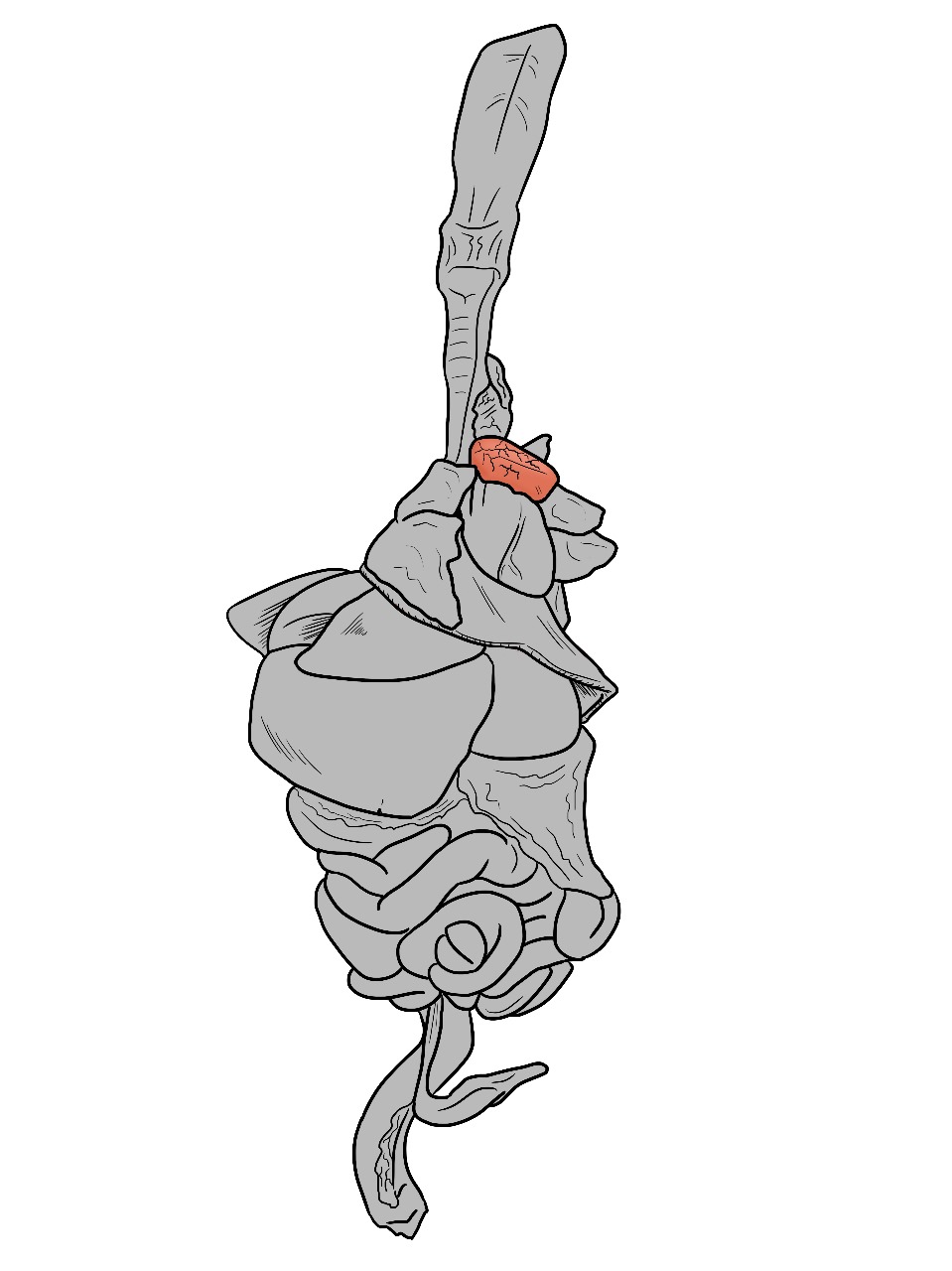

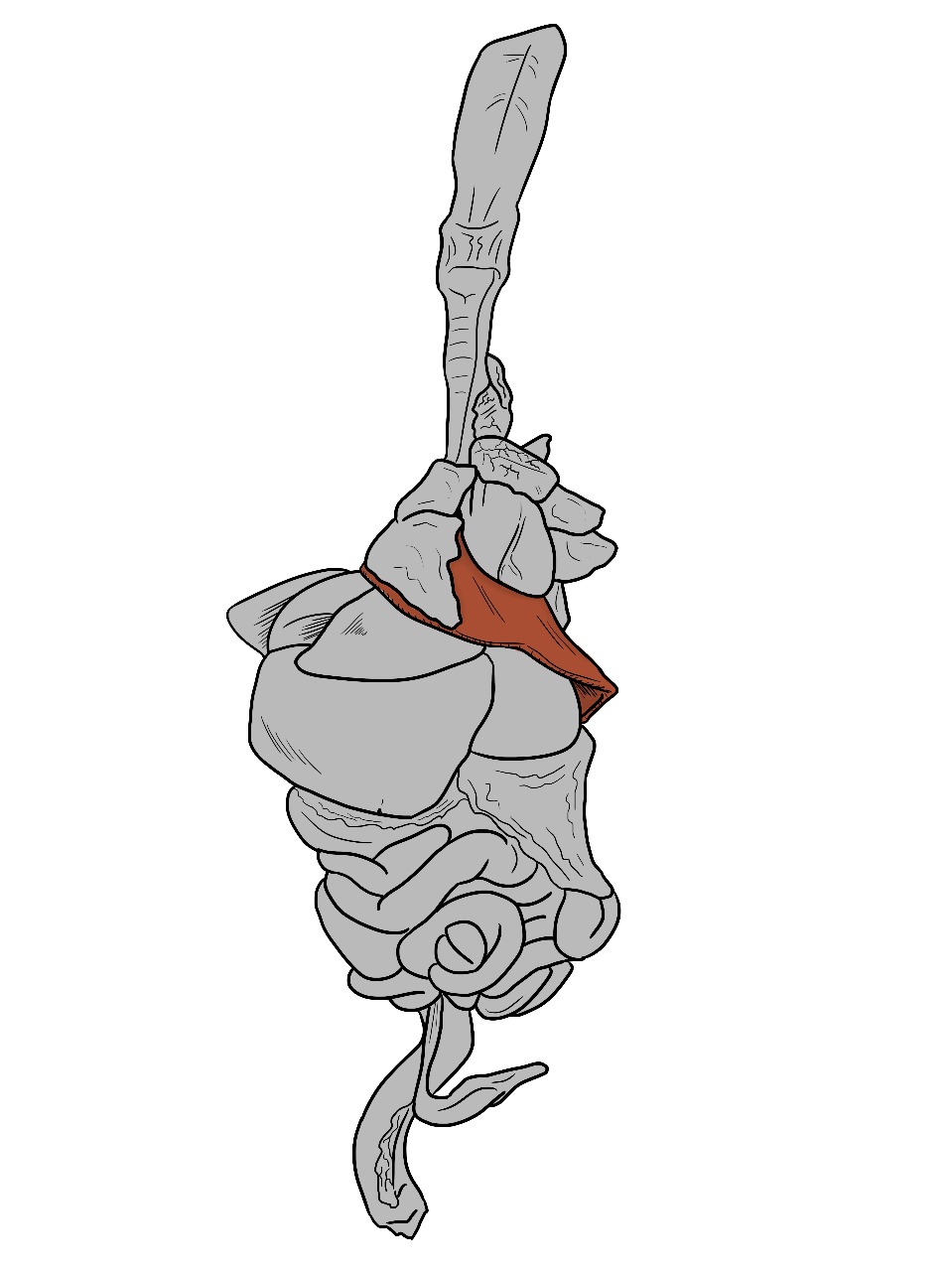

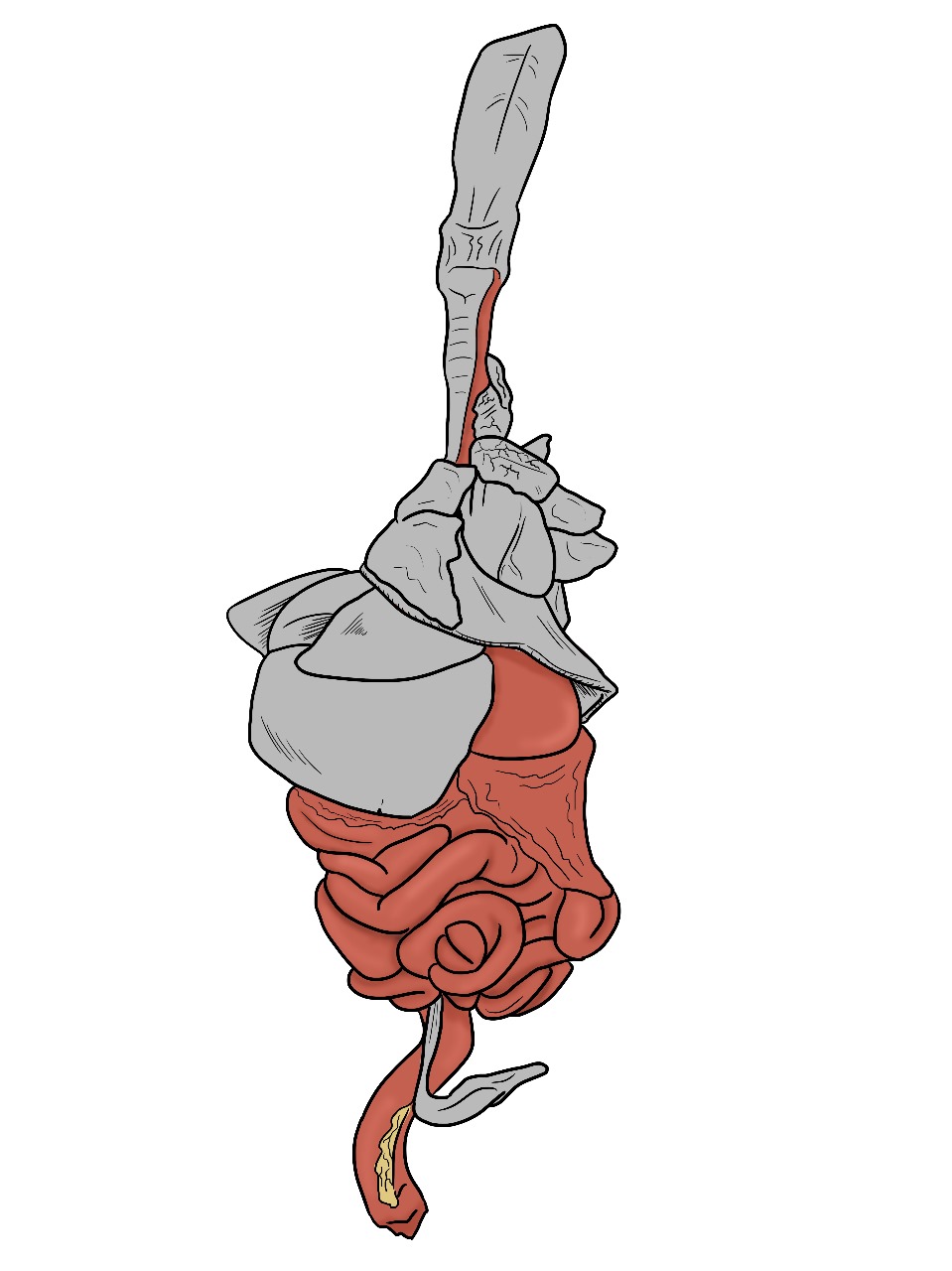

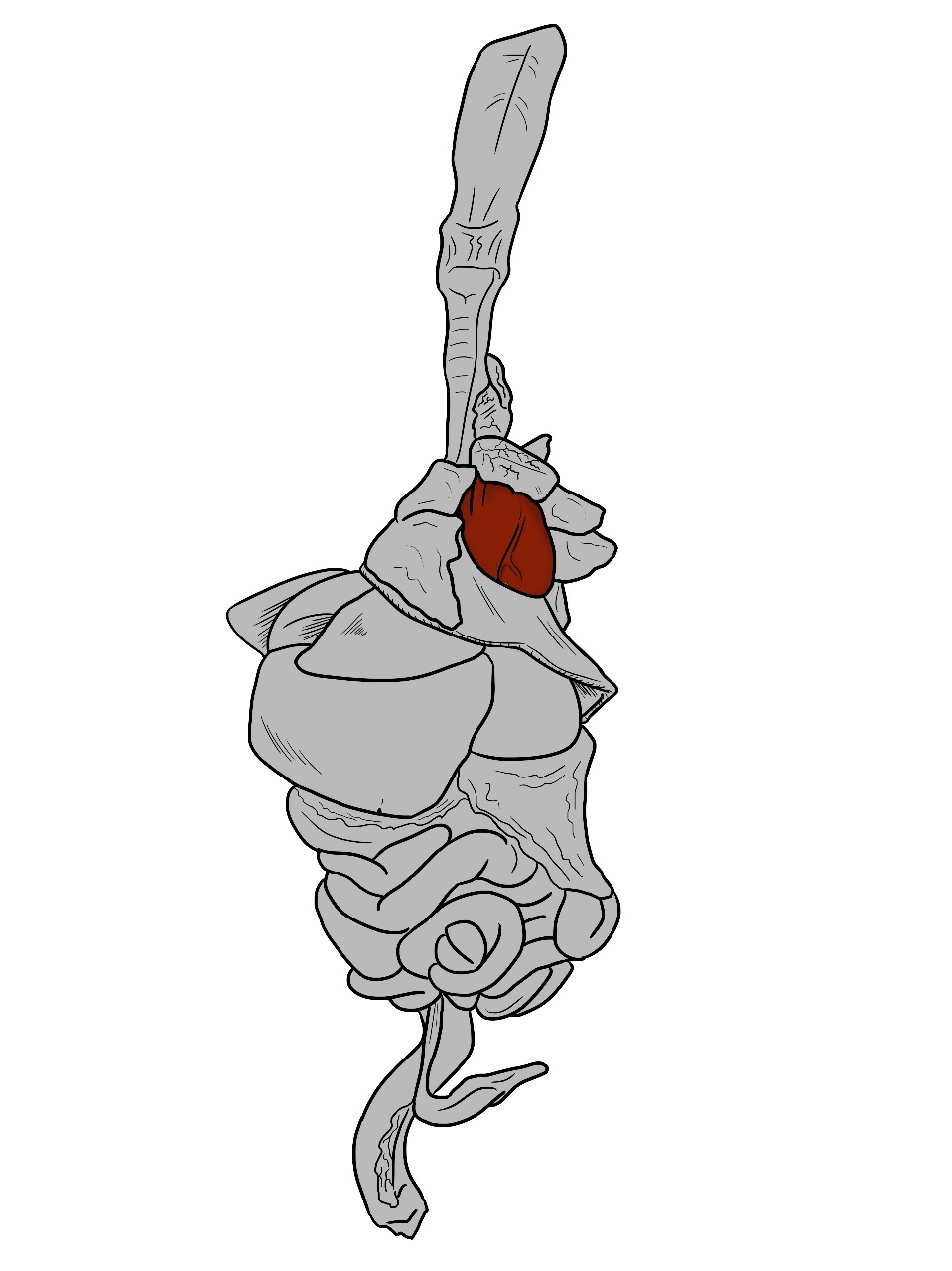

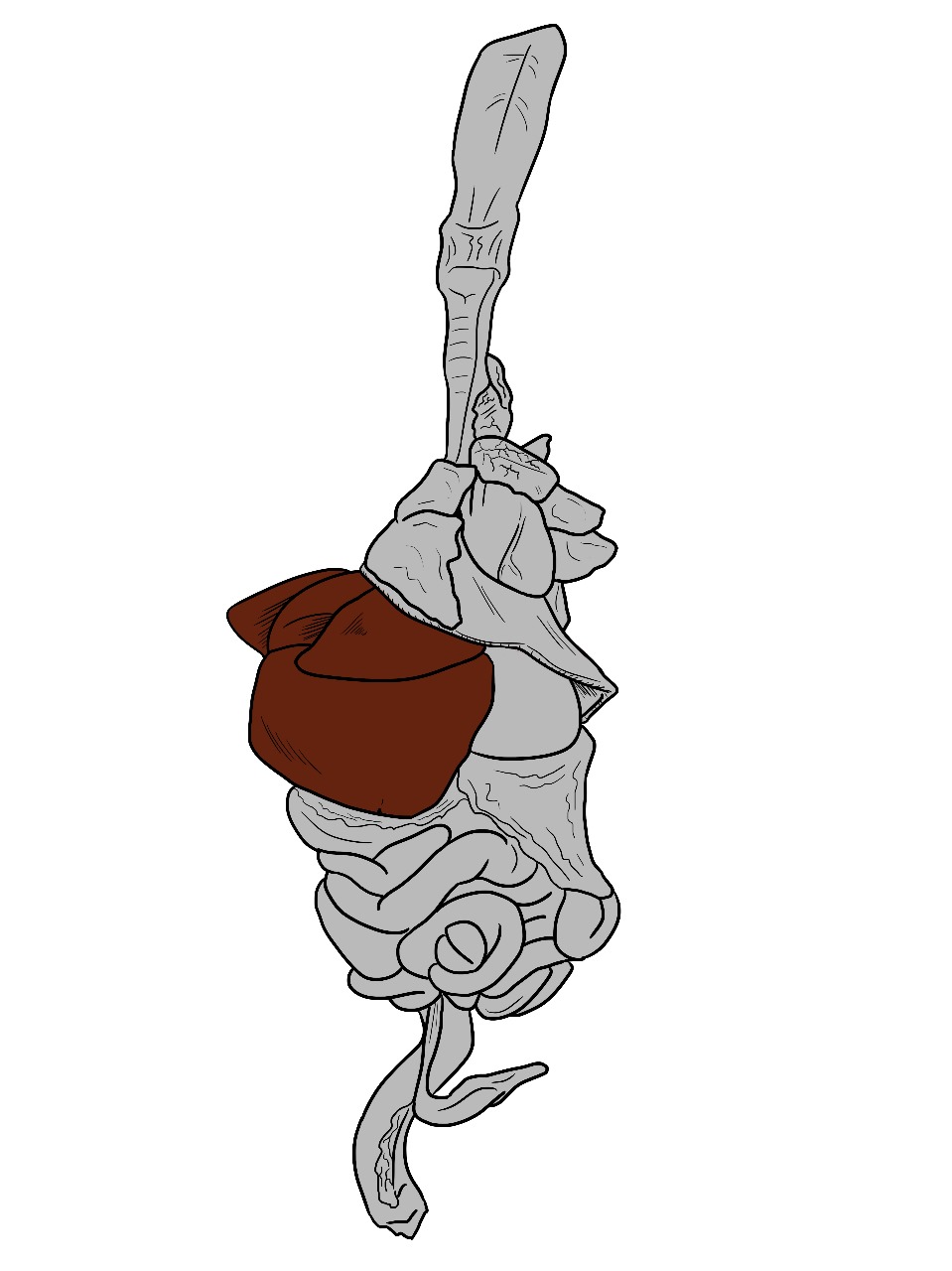
**

**
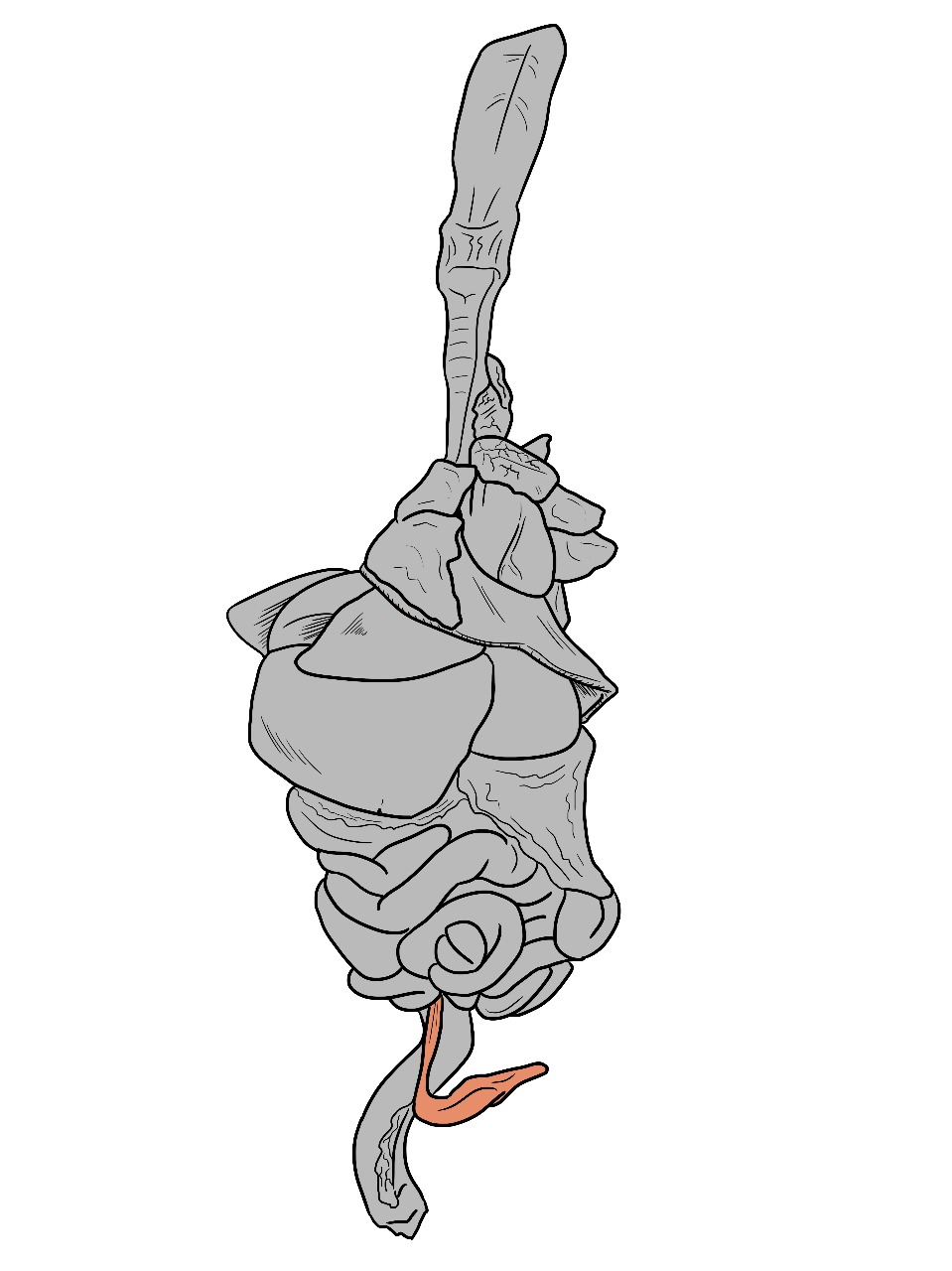
**

**
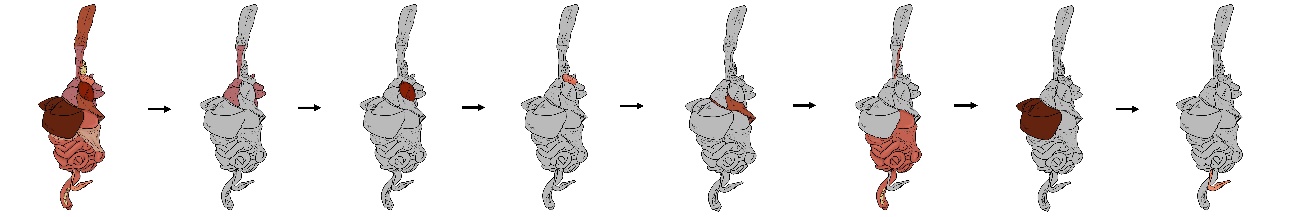
**
